# First-in-Human Study of 23ME-00610, an Antagonistic Antibody for Genetically Validated CD200R1 Immune Checkpoint, in Participants with Advanced Solid Malignancies

**DOI:** 10.1158/2767-9764.CRC-24-0568

**Published:** 2025-01-15

**Authors:** Shivaani Kummar, Albiruni Abdul Razak, Scott Laurie, Dylan M. Glatt, Sariah Kell, Anh N. Diep, Maike Schmidt, Clifford Hom, Chris German, Suyash S. Shringarpure, Sophia R. Majeed, Drew Rasco

**Affiliations:** 1 Knight Cancer Institute, Oregon Health Sciences University, Portland, Oregon.; 2 Princess Margaret Cancer Centre, Toronto, Canada.; 3 The Ottawa Hospital, Ottawa, Canada.; 4 23andMe Therapeutics, South San Francisco, California.; 5 23andMe, Sunnyvale, California.; 6 The START Center for Cancer Care, San Antonio, Texas.

## Abstract

**Purpose:**

In this phase 1 portion of a first-in-human phase 1/2a study (NCT05199272), 23ME-00610 was evaluated in participants with advanced solid malignancies to determine its safety, tolerability, pharmacokinetics (PK), and pharmacodynamics (PD). Exploratory biomarkers were evaluated to examine potential correlates of efficacy and safety.

**Patients and Methods:**

Eligible participants (≥18 years) were administered 23ME-00610 intravenously every 3 weeks (Q3W) using an accelerated titration design followed by a traditional 3 + 3 design, with an initial dose level of 2 mg.

**Results:**

Twenty-eight participants were enrolled across seven cohorts and received a median of four cycles of 23ME-00610. No treatment-related serious adverse events (AE) were observed, and the maximum tolerated dose was not reached. Overall, the PK of 23ME-00610 was linear and dose proportional for doses ≥60 mg, with a median terminal half-life of 13 days at 1,400 mg. Peripheral saturation of CD200R1 was observed for doses ≥60 mg. Immune-related AEs, including rash, pruritus, and hypothyroidism, were predicted by phenome-wide association studies and observed for doses ≥60 mg. A confirmed partial response was observed in a participant with well-differentiated pancreatic neuroendocrine cancer whose tumor was among those with the highest tumor CD200 expression.

**Conclusions:**

23ME-00610 has mild-to-moderate on-target AEs and PK/PD consistent with tumor target saturation and dosing every 3 weeks. The trend for clinical benefit in participants with tumor CD200 expression suggests that 23ME-00610 inhibits CD200R1 signaling and may reverse CD200-mediated immune evasion. Based on PK/PD, safety, and preliminary antitumor activity, 1,400 mg Q3W was selected as the dose for further study.

**Significance:**

Genome-wide association studies (GWAS) of the 23andMe genetic database identified CD200R1 as a promising therapeutic target for cancer. This phase 1 study of 23ME-00610, a CD200R1 antagonist IgG1, showed acceptable safety and tolerability, PK supporting Q3W dosing, and PD and preliminary clinical activity supporting an initial recommended phase 2 dose of 1,400 mg.

## Introduction

CD200R1 is a key regulator of immune cell function and is critical for maintaining immune tolerance (1–6). This inhibitory receptor is expressed predominantly on T cells, myeloid cells (including neutrophils, eosinophils, mast cells, and macrophages), and NK cells (7–12). Its corresponding and only known ligand in humans, CD200, is expressed on a broad range of cell types, including endothelial cells, neurons, thymocytes, and immune cell populations such as B cells and follicular dendritic cells (1, 8, 13, 14). CD200 is also highly expressed on tumor cells and some stromal cells in the tumor microenvironment in several hematologic and solid tumor malignancies (15, 16). An *in silico* screen for shared genome-wide association study signals across hundreds of phenotypes identified pleiotropic opposing effects on the lifetime risk for cancer and autoimmune disease on gene variants for CD200R1, CD200, and Dok2 (10, 12). A similar genetic pattern of opposing risk for cancer and autoimmune disease has been observed for clinically validated immunotherapy targets, including CTLA4 (10). Thus, targeting CD200R1 for breaking CD200/CD200R1 immune tolerance in cancer presents an opportunity to target multiple immune cell populations in addition to T cells in the tumor (17), such as myeloid and NK cells, which has been shown to be a promising approach in preclinical research (18, 19).

23ME-00610 is a first-in-class, high-affinity (*K*_*D*_ < 0.1 nmol/L), effectorless, fully humanized, IgG1 mAb that binds to the CD200R1 immune checkpoint and prevents its binding to CD200 (10). Based on nonclinical studies, 23ME-00610 leads to restoration of immune cell activity, including in T cells, and mediates immune cell killing of CD200-expressing tumor cells (10, 20). 23ME-00610 was well tolerated in monkeys, had a half-life of 10 to 13 days, which supported an every 3 weeks (Q3W) dose schedule in the first-in-human (FIH) study, and showed minimal risk for cytokine release based on *in vitro* cytokine release assays (21). Furthermore, no hazards were identified with an anti-CD200R1 surrogate antibody in repeat-dose toxicology studies in monkeys at levels ≤100 mg/kg, leading to a no observed adverse effect level (NOAEL) of 100 mg/kg (21, 22). Exposure effect modeling was conducted to determine the 2 mg MABEL starting dose and the projected efficacious dose of ≥600 mg (10, 20, 21). Antitumor activity at doses ≥600 mg was predicted based on a pharmacologically relevant tumor cell–killing assay and clinical serum pharmacokinetic (PK) target corresponding to a predicted cycle 1 trough tumor concentration >EC_90_ (21).

In this study, we report the results of the phase 1 dose escalation portion of the FIH study of 23ME-00610 conducted in participants with advanced solid malignancies. A 3 + 3 study design was used, except for the lowest dose levels, which were accelerated titration (single-patient) cohorts. The primary objectives were to evaluate the safety and tolerability of 23ME-00610 and to determine the maximum tolerated dose (MTD) and/or the recommended phase 2 dose. Additional objectives were to evaluate immunogenicity, characterize the PK profile of single and multiple doses, explore the pharmacodynamic (PD) effects, and evaluate preliminary clinical activity of 23ME-00610. Based on analogous studies with other checkpoint inhibitors that have shown a relationship between tumor pathway expression [e.g., PD-(L)1] and clinical response to pembrolizumab (23), nivolumab (24), and atezolizumab (25), baseline assessments for tumor CD200R1 and CD200 expressions were performed to evaluate potential correlates with efficacy. This study also consented and genotyped participants using a single saliva sample, which was used to deliver real-time, personalized genetic information to participants who provided consent. Because polygenic risk scores (PRS) can identify patients at higher risk for developing immune-related AEs (irAEs), particularly skin autoimmunity (26), thyroid autoimmunity (27), and ulcerative colitis (28), and irAEs are higher for patients receiving clinical benefit from immune checkpoint inhibitor therapy (26–28), exploratory PRS analysis, which quantitates an individual’s genetic risk for a condition (29), was conducted for each participant to determine whether the trial participants had similar genetic features and GWAS signals as was seen from the *in silico* analysis that identified CD200R1 as a drug target.

## Materials and Methods

### Study design

This was an open-label, phase 1, multicenter study of 23ME-00610 (ClinicalTrials.gov; NCT05199272). Protocol and informed consent were approved by each study center’s Institutional Review Board, complied with the Declaration of Helsinki, and each participant provided written informed consent before study participation. Eligible participants were at least 18 years of age, had a histologically diagnosed locally advanced (unresectable) or metastatic solid cancer that had progressed after all available standard therapy for the specific tumor type or no further standard therapy existed, and had an Eastern Cooperative Oncology Group performance status of 0 or 1 and RECIST measurable or evaluable disease. Key exclusion criteria included presence of active autoimmune disease that required immunosuppressive treatment in the last 2 years, history of grade ≥3 immune-mediated toxicity related to prior immunotherapy that led to discontinuation, or uncontrolled or symptomatic central nervous system metastases and/or carcinomatous meningitis.

Consented eligible participants were enrolled into sequential cohorts of increasing doses of 23ME-00610 administered by i.v. infusion Q3W infused over 30 minutes with a 21-day dose-limiting toxicity observation period following administration of the first dose. Because this was a FIH study, a formal power calculation was not required, there was no blinding, and participants were not randomized. Participants were administered fixed doses of 23ME-00610, as analyses of body weight–based and fixed doses of investigational therapeutic mAb cancer therapies have shown no clinically meaningful differences in exposures (30). The study design is illustrated in Supplementary Fig. S1. The dose escalation consisted of an accelerated titration phase for the first two cohorts (2 and 6 mg), in which for each cohort 1 participant was enrolled, followed by a traditional “3 + 3” design beginning in cohort 3 (for planned doses of 20, 60, 200, 600, and 1,400 mg). Additional participants (up to 12, including the first 3–6 initially enrolled during dose escalation) were enrolled in a PK/PD backfill cohort at the 600 and 1,400 mg dose levels. Intraparticipant dose escalation was permitted with approval of the medical monitor to optimize the number of participants treated at a potentially clinically relevant dose. Provided the participant had not experienced a grade 3 or higher drug-related AE at the assigned dose, they could be escalated to a higher dose that was determined to be sufficiently tolerable by the safety review committee (SRC).

Before dose escalation, the safety, tolerability, and available PK and PD data at each dose level, including data from ongoing participants in prior cohorts, were evaluated by the SRC. The SRC, at minimum, comprised the participating principal investigators, the medical monitor, and a drug safety representative.

### Assessments

#### Safety

Safety assessments included vital signs, 12-lead ECG, physical examination, and clinical laboratory assessments (thyroid function, morning cortisol, hematology, chemistry, coagulation, urinalysis, and pregnancy testing), Eastern Cooperative Oncology Group performance status and AEs were collected before dosing, during the dosing period, and through the 90-day follow-up period. AEs were graded according to the NCI–Common Terminology Criteria for AEs version 5.0 and were coded using the Medical Dictionary for Regulatory Activities version 24.1. irAEs were defined as clinically significant AEs occurring in any organ that were associated with exposure to 23ME-00610, were of unknown etiology, and were consistent with an immune-related mechanism (31). Management of irAEs and guidelines for dose modification were based on the American Society of Clinical Oncology clinical practice guidelines (32).

#### Efficacy

RECIST 1.1 (33) was used in the assessment of tumor response endpoints (including target and nontarget lesion determination) by local investigator assessment. iRECIST (34) was used by investigators to assess tumor response and progression for treatment decisions. All participants had the extent of their disease assessed by a staging CT/MRI scan at baseline, and subsequent scans were required every 8 weeks (±10 days) while on treatment for participants with measurable disease (Supplementary Fig. S2).

#### PK

For PK analyses, serial blood samples were collected predose and up to day 21 postdose on cycles 1 and 4. Sparse PK samples were collected in every cycle. Serum concentrations of 23ME-00610 were determined by Precision for Medicine using a validated Meso Scale Discovery–based ligand-binding assay with a lower limit of quantification of 0.25 μg/mL. PK parameters [e.g., AUC, maximum or peak concentration (C_max_), time of maximum observed concentration (T_max_), and half-life (T_1/2_)] were estimated by noncompartmental methods using Phoenix WinNonlin (RRID: SCR_024504; Certara, version 8.3).

#### PD

Blood samples were collected for evaluation of PD biomarkers (target engagement, soluble pathway components, and cytokines to assess immune cell activation). Target engagement was assessed using assays that were developed to measure CD200R1 receptor occupancy (RO), free soluble CD200R1, and total soluble CD200R1. Samples for these assessments were collected predose before cycles 1 and 2 and several timepoints postdose after cycles 1 and 2. Additional predose samples were collected at cycles 3, 4, and 6 for the soluble assays. All PD sample collections had time-matched PK sample collections.

The RO assay measured 23ME-00610 bound to CD200R1 on clusters of differentiation 4 (CD4^+^) T cells and neutrophils in blood, in which target engagement in the qualitative assay is defined as RO >60% being occupied and less than 60% not being occupied on each population.

The free soluble CD200R1 assay was quantitative and measured unbound soluble CD200R1, and target engagement was defined as a reduction of baseline levels to lower levels, including below the lower limit of quantitation. The total soluble CD200R1 assay was also quantitative and measured both unbound and bound soluble CD200R1. CD200R1 target engagement was deduced by an increase in detectable total soluble CD200R1 relative to baseline levels. The quantifiable range of the standard curve was 312 to 10,000 pg/mL in the free assay and 312 to 25,000 pg/mL in the total assay.

#### CD200 and CD200R1 IHC assays

The expression of CD200R1 and CD200 was evaluated by IHC in formalin-fixed paraffin-embedded (FFPE) archival or baseline tumor tissue samples collected from study participants (Supplementary Fig. S3A and S3B). Four- to five-mm-thick sections of FFPE archival or freshly biopsied tumors were stained with Ventana CD200 (SP517) and Ventana CD200R1 (SP503) assays developed by Ventana. Visual examination of CD200 expression was conducted by board-certified anatomic pathologists, who scored membrane-associated CD200 with an H-score assessing both intensity and percentage of CD200-positive tumor cells (range 0 to 300; Supplementary Fig. S3C). Immune cell and tumor cell CD200R1 expression was reviewed and scored as the percentages of positive cells within the immune cell population and tumor cell population, respectively (Supplementary Fig. S3C).

### Genetic analyses and PRS calculation

To characterize participant germline genetics, participants enrolled in the 23ME-00610 clinical trial were separately consented for genotyping, were evaluated using the saliva-based 23andMe genotyping platform, and were informed that they could access their 23andMe data on the Personal Genome Service platform. Genotype data of trial participants of European descent [*N* = 14 (50.0%)] were imputed using the Haplotype Reference Consortium panel (35). Data from the 23andMe, Inc. Research Cohort (36) of European ancestry were used to construct PRSs for a prespecified set of immune-mediated (vitiligo, hypothyroidism, mosquito bite itching, Hashimoto’s disease, chronic hives, allergies, rosacea, psoriasis, eczema, and asthma) and cancer phenotypes (colorectal cancer, prostate cancer, breast or ovarian cancer, and melanoma; Supplementary Fig. S4). Phenotypes were selected based on a combination of literature precedent for association with clinical outcomes as well as those that are most adequately powered from the 23andMe health and survey database. The reference population for *Z*-score analysis consists of research participants of European ancestry from the 23andMe database.

### Statistical analyses

Study data were summarized using descriptive statistics for disposition, demographics, baseline disease characteristics, safety, and clinical activity. Tabular summaries were grouped by dose level/cohort and overall. Categorical variables were summarized by frequency distribution (i.e., number and percentage of participants), and continuous variables were summarized by descriptive statistics (i.e., mean, SD, median, minimum, and maximum).

PK parameters were summarized using descriptive statistics by cohort, dose level and cycle. Dose proportionality was assessed by comparing PK parameters [e.g., area under the concentration–time curve (AUC) and maximum serum concentration (C_max_)] across the dose cohorts using a power model to evaluate the population mean slope based on its 90% confidence interval.

PD measures (target engagement, soluble biomarkers, and cytokines) were summarized using descriptive statistics for 23ME-00610 by cohort and/or dose level over time.

Immunogenicity data, including prevalence (ADA-positive at baseline) and incidence (sum of treatment-induced and treatment-boosted ADA) of ADA to 23ME-00610 were summarized. The frequency and percentage of participants with positive and negative immune response results were summarized for each assessment time and by cohort and dose level.

Participants who did not have postbaseline tumor assessments were assigned a best overall response (BOR) of not evaluable (NE). Participants who experienced clinical progression before the first postbaseline tumor assessment were assigned a BOR of progressive disease (PD). For progression-free survival per RECIST v1.1, progression-free survival per iRECIST, duration of response per RECIST v1.1, and duration of response per iRECIST, these participants were considered as PD, and the PD date was the date of clinical PD.

The percentage of participants with archival tissue evaluable for the exploratory biomarkers, CD200 and CD200R1 expression levels, were summarized with semi-quantitative scores reported.

### Data availability

The data generated from this study are publicly available on the ClinicalTrials.gov results database at NCT05199272, per FDAAA 801 and the Final Rule.

## Results

### Patient population and disposition

Between January 5, 2022, and January 31, 2023, 28 participants with locally advanced (unresectable) or metastatic solid malignancies were enrolled. Data cut-off for this analysis was October 2, 2024. Baseline characteristics for enrolled participants are described in Table 1. Based on age, sex, and race/ethnicity distribution, the enrolled participants were representative of the advanced solid malignancy population for the United States and Canada (Supplementary Table S1). Participants had received a median of three lines of anticancer therapies before study entry (range 1–9) and 53.6% (*N* = 15) had received prior anti–PD-(L)1 or anti-CTLA4 immunotherapy. All 28 participants received ≥1 dose of 23ME-00610 [median (range): 4 (1–31)] in 7 dose-level cohorts ranging from 2 to 1,400 mg (Table 2).

**Table 1:** Baseline patient and disease characteristics

Characteristic	Total population, *N* = 28
Median age, years (range)	62.0 (21, 80)
Female sex, *n* (%)	14 (50.0%)
Race, *n* (%)	
White	22 (78.6%)
Black or African American	1 (3.6%)
Asian	2 (7.1%)
American Indian or Alaska Native	1 (3.6%)
Unknown/other	2 (7.1%)
Hispanic or Latino ethnicity, *n* (%)	6 (21.4%)
Enrolled in the United States, *n* (%)	23 (82.1%)
Enrolled in Canada, *n* (%)	5 (17.9%)
ECOG performance status, *n* (%)	
0	11 (39.3%)
1	17 (60.7%)
Median number of prior anticancer therapies, *n* (range)	3.0 (1, 9)
Median time from initial diagnosis, months (range)	29.3 (3.5, 181.4)
Prior immunotherapy, *n* (%)	15 (53.6%)
Primary cancer type, *n* (%)	
Colorectal	5 (17.9%)
Pancreatic	4 (14.3%)
Neuroendocrine	3 (10.7%)
Esophageal	2 (7.1%)
Melanoma	2 (7.1%)
Sarcoma	2 (7.1%)
Breast	1 (3.6%)
Osteosarcoma	1 (3.6%)
Prostate	1 (3.6%)
Endometrial	1 (3.6%)
Other	6 (21.4%)

Abbreviations: ECOG, Eastern Cooperative Oncology Group.

**Table 2:** 23ME-00610 treatment exposure and AE summary

Dose level^b^	2 mg (*N* = 1)	6 mg (*N* = 1)	20 mg (*N* = 3)	60 mg (*N* = 4)	200 mg (*N* = 3)	600 mg (*N* = 8)	1,400 mg (*N* = 8)	Total (*N* = 28)
Number of doses received								
Median (range)	11	11	3 (2–15)	3 (3–6)	6 (6–28)	2.5 (1–31)	3.5 (1–15)	4 (1–31)
Treatment duration (days)								
Median (range)	229	231	51 (41–338)	73 (63–124)	134 (126–690)	53 (29–635)	70 (24–335)	93.5 (24–690)
Overview of AEs, *n* (%)								
All TEAEs	1 (100.0%)	1 (100.0%)	3 (100.0%)	4 (100.0%)	3 (100.0%)	8 (100.0%)	7 (87.5%)	27 (96.4%)
Any irAE	0	0	0	1 (25.0%)	2 (66.7%)	5 (62.5%)	4 (50.0%)	12 (42.9%)
TRAEs	1 (100.0%)	0	2 (66.7%)	2 (50.0%)	3 (100.0%)	6 (75.0%)	5 (62.5%)	19 (67.9%)
TRAEs leading to D/C	0	0	0	0	0	1 (12.5%)	0	1 (3.6%)
Grade ≥3 AEs^a^	0	0	1 (33.3%)	2 (50.0%)	1 (33.3%)	5 (62.5%)	3 (37.5%)	12 (42.9%)
Treatment-related grade 3 AEs^a^	0	0	0	0	0	2 (25.0%)	1 (12.5%)	3 (10.7%)
23ME-00610–related AEs of any grade observed ≥5% of participants and including all **irAEs**								
Pruritus/urticaria	1	0	0	0	**1**	**2**	0	4 (14.3%)
Rash	0	0	0	0	**1**	**2**	**1**	4 (14.3%)
Hypothyroidism/increased TSH	0	0	0	**1**	0	**1**	**2**	4 (14.3%)
Nausea	0	0	0	0	0	2	2	4 (14.3%)
Fatigue	0	0	0	1	2	0	0	3 (10.7%)
Arthralgia	0	0	1	0	0	1	1	3 (10.7%)
Headache	0	0	0	1	1	1	0	3 (10.7%)
Cough	0	0	0	0	0	0	2	2 (7.1%)
Immune-related reaction	0	0	0	0	0	0	**1**	1 (3.6%)

The bold and underlined values indicate those AEs that were determined to be immune-related AEs (irAEs).

Abbreviations: D/C, discontinuation; TEAE, treatment-emergent adverse event; TSH, thyroid-stimulating hormone.

a There were no grade 4 or 5 AEs.

b Summarized based on the initial dose level the patient received.

At data cut-off, 27 of the 28 participants (96.4%) had discontinued treatment (Supplementary Table S2). One participant (3.6%), who received a 600 mg dose, discontinued due to a grade 3 treatment-related AE (TRAE) of maculopapular rash, and 26 participants (92.9%) discontinued due to disease progression. Twelve participants (42.9%) had received at least six cycles of 23ME-00610 treatment, and three participants (10.7%) had received at least 13 cycles. One participant, a 49-year old female with stage IIIA endometrial adenocarcinoma (microsatellite stable), progressed after cycle 13 and continued treatment through cycle 14. The participant discontinued treatment 71 days after progression. No other participants were treated beyond progression.

### Safety and tolerability

All 28 participants were included in the safety analysis. The overall AE summary is presented in Table 2. Participants were enrolled in a dose escalation of doses from 2 to 1,400 mg, and the MTD was not reached. The median duration of time participants on 23ME-00610 treatment was 93.5 days (maximum of 690 days). One dose-limiting toxicity was observed in a patient enrolled at the 600 mg dose level PK backfill cohort; this patient experienced a grade 3 maculopapular rash that resolved with supportive care and did not receive additional drug. Overall, 27 participants (96.4%) experienced at least one treatment-emergent AE irrespective of the relationship to 23ME-00610. Most of these events were grade 1 or 2 in severity. There were no grade 4 or 5 treatment-emergent AEs. TRAEs occurred in 19 participants (67.9%), and most events were grade 1 or 2 in severity (Table 2). Three participants (10.7%) experienced grade 3 TRAEs: maculopapular rash (600 mg), elevated alkaline phosphatase (1,400 mg), and elevated creatine phosphokinase (600 mg), all appearing in cycle 1. The laboratory parameter elevations were asymptomatic. Seven serious AEs (SAE) occurred in six participants (21.4%; Supplementary Table S3), and no SAEs were observed more than once. There were no SAEs considered related to 23ME-00610 as assessed by an investigator; these events were attributed to other known causes. irAEs were observed in 12 participants (42.9%; Table 2), occurred at doses ≥60 mg, and were primarily grade 1 or 2 in severity. The most frequently occurring irAEs were hypothyroidism (*n* = 4, 14.3%), rash (*n* = 4, 14.3%), and pruritis/urticaria (*n* = 4, 14.3%). Six participants (21.4%) had at least one irAE that appeared within the first cycle of treatment, and seven participants (25.0%) had at least one irAE that appeared after cycle 1.

### PK and target engagement

#### PK

**Figure 1:**
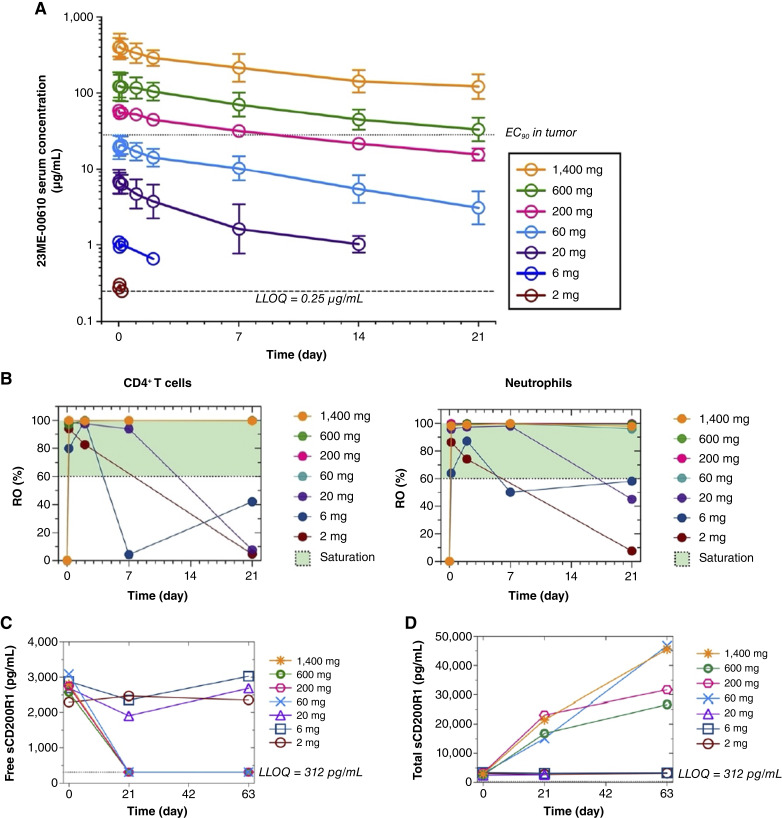
Single-dose serum PK and peripheral target engagement for 23ME-00610 in patients with cancer by dose level. Sample size for participants with evaluable data by dose cohorts: 1,400 mg (*N* = 8), 600 mg (*N* = 8), 200 mg (*N* = 3),60 mg (*N* = 4), 20 mg (*N* = 3), 6 mg (*N* = 1), and 2 mg (*N* = 1). **A,** Geometric mean and geometric SD for cycle 1 serum 23ME-00610 concentration–time data by dose level. Below limit of quantitation (BLQ) values were set to missing, and data were plotted only if more than half of the evaluable data was quantifiable [greater than the lower limit of quantitation (LLOQ)] and not missing. EC_90_ in tumor PK target (dotted reference line) based on *in vitro* tumor-killing assay and 10% tumor-to-serum partition. **B,** Median RO by dose level for cycle 1. The RO assay is qualitative and measures 23ME-00610 bound to the target receptor CD200R1 on the target cell of interest. At least 60% RO (shaded in green) is considered saturated, in which less than 60% RO is considered not saturated. **C,** Median-free soluble CD200R1 by dose level from cycle 1 day 1 predose to cycle 4 day 1 predose (63 study days). The assay lower limit of detection (following a 20-fold minimum required dilution) is 312 pg/mL (dotted reference line). BLQ values were plotted at the LLOQ. All patients for dose levels ≥60 mg were BLQ at day 21 and day 63. **D,** Median total soluble CD200R1 (sum of free soluble CD200R1 and 23ME-00610–bound soluble CD200R1) by dose level from cycle 1 day 1 predose to cycle 4 day 1 predose (63 study days). The assay lower limit of detection (following a 20-fold minimum required dilution) is 312 pg/mL (dotted reference line).

All 28 participants had evaluable PK data at the time of data cut-off. 23ME-00610 PK was linear and dose proportional for doses from 60 to 1,400 mg (Fig. 1A), and exposure parameters (e.g., C_max_, AUC_0-21D_, and AUC_inf_) increased ∼2-fold for repeat dosing Q3W (21). 23ME-00610 clearance was faster at doses <60 mg Q3W, likely due to incomplete saturation of CD200R1. For doses in the linear range, 23ME-00610 had a median cycle 1 T_1/2_ of 11 to 13 days, and the median cycle 1 T_1/2_ was 13 days at 1,400 mg (21). The 1,400 mg dose achieved the PK target (i.e., C_trough_ > predicted EC_90_ in tumor) for all participants (Fig. 1A). There was no evidence of antidrug antibody (ADA) formation with repeated administration of 23ME-00610 (Supplementary Table S4).

#### Target engagement

The binding of 23ME-00610 to the target receptor CD200R1 on clusters of differentiation 4 (CD4^+^) T cells and neutrophils was evaluated in all participants (Fig. 1B). Sustained saturation of peripheral RO on CD4^+^ T cells and neutrophils was observed for doses ≥60 mg throughout the first cycle of treatment with 23ME-00610. RO was dose-responsive for doses <60 mg. For example, saturated RO was observed at 4 and 48 hours after 23ME-00610 administration for 2 and 6 mg, respectively, and for up to 7 days after administration for 20 mg.

Free (unbound) and total (sum of unbound and bound) soluble CD200R1 was measured from participant plasma samples to further assess 23ME-00610 target engagement (Fig. 1C and D). As expected, the baseline plasma concentration of free and total soluble CD200R1 were similar; the median baseline free level in participants was 2,766 pg/mL (range 2,175–5,201 pg/mL), and the median baseline total level was 2,960 pg/mL (1,870–4,433 pg/mL). Plasma concentrations of free soluble CD200R1 decreased immediately following 23ME-00610 administration [i.e., the first timepoint postdose (4 hours)] for all dose levels; however, the durability of suppressing free soluble CD2001 to undetectable levels was dose-dependent. Free soluble CD200R1 was undetectable at all postdose sample collections for doses ≥60 mg, whereas free soluble CD200R1 returned to baseline in a dose-dependent manner for doses <60 mg. In line with 23ME-00610 binding to soluble CD200R1, increasing doses of 23ME-00610 led to higher levels of total soluble CD200R1 postdose. Increases in total CD200R1 level were similar for doses ≥60 mg, accumulating and plateauing at ≥ 10,000 pg/mL by Day 21 (i.e., C2D1) for the 600 and 1,400 mg cohorts, whereas 23ME-00610 doses <60 mg resulted in no change from baseline for total soluble CD200R1. Taken altogether, 23ME-00610 doses ≥60 mg saturate peripheral soluble CD200R1, and 23ME-00610 doses <60 mg show dose-dependent binding and are unable to saturate soluble CD200R1. Collectively, based on paired PK data, 23ME-00610 serum concentrations ≥ ∼1.5 μg/mL (minimum cycle 1 C_trough_ for 60 mg) are sufficient to saturate peripheral CD200R1.

### Efficacy

**Figure 2:**
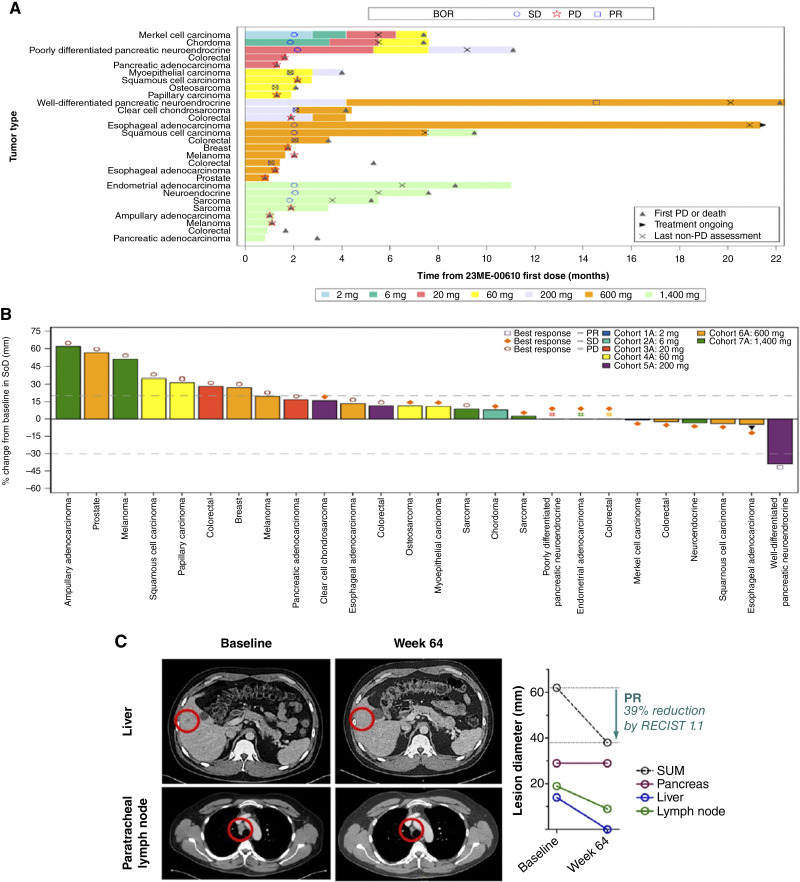
Patients were scanned and assessed by RECIST v1.1 approximately every 8 weeks. **A,** Swimmer plot showing the treatment duration and BOR for enrolled patients across all dose levels. BOR for PD (star), SD (circle), and PR (square) is shown. Additional annotations for first PD or death (upward gray triangle), the last non-PD assessment (X mark), and treatment ongoing (right-facing black triangle) are included. Each color represents the cohort/dose level. Eight patients that initially received doses ≤600 mg had their dose escalated during treatment. **B,** Waterfall plot showing the %change in baseline target lesion SoD and BOR for enrolled patients across all dose levels. Each color represents the cohort/dose level initially received. Best response is noted for PD (circle), SD (diamond), and PR (square). One patient remains on treatment as of the data cut date is noted with a black triangle. *N* = 2 patients were not evaluable due to missing postbaseline assessment; thus, only *N* = 26 patients with BOR data per RECIST v1.1. **C,** Baseline and week 64 scans and target lesion diameter summary for the patient with a well-differentiated pancreatic neuroendocrine tumor with confirmed PR. Data as of October 2, 2024. SoD, sum of diametres.

All 28 participants were evaluable for treatment response. There was one RECIST response [partial response (PR); 3.6%], and 13 of the 28 participants (46.4%) had stable disease (SD; Fig. 2A; Supplementary Table S5). The participant with a RECIST response had well-differentiated pancreatic neuroendocrine cancer with no PD-L1 expression, was microsatellite stable, and did not have high tumor mutational burden. Enrolled at 200 mg and with dose escalation to 600 mg, the participant had a 39% reduction in the sum of diameters of target lesions (Fig. 2B), including complete disappearance of a liver target lesion and significant reduction of the lymph node lesion (>50%) by week 64 (Fig. 2C); moreover, the participant remained on study for approximately 20 months before disease progression. One participant with stage IIIB esophageal adenocarcinoma enrolled at 600 mg had SD for more than 20 months, resolution of abdominal malignant ascites (Supplementary Fig. S2), and remains on study receiving 600 mg 23ME-00610 as of data cut-off date. Microsatellite instability and tumor mutational burden were not tested for this patient; however, the tumor had a PD-L1 combined positivity score of 2%. Of the 19 participants treated at the 23ME-00610 projected efficacious dose ≥600 mg, including three participants whose dose was escalated from 200 mg, approximately half of the participants (9/19, 47.4%) had a BOR of SD or PR, and five participants (26%) had meaningful clinical benefit (PR or SD > 6 months). Moreover, the duration of 23ME-00610 treatment for participants with a BOR of PR or SD was longer on average (median: 211 days) than the duration of 23ME-00610 exposure for the full *N* = 28 participant cohort (Supplementary Table S6).

### Exploratory biomarker analyses

**Figure 3:**
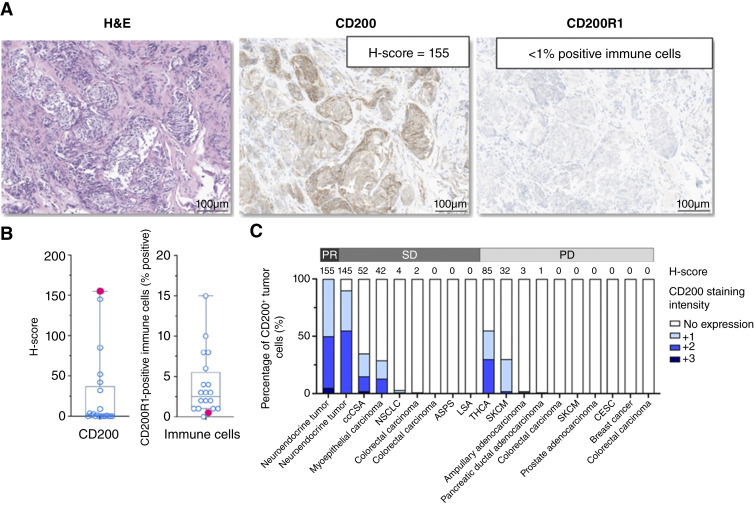
CD200 and CD200R1 expression in participant tumors. **A,** Representative photomicrographs of H&E, CD200, and CD200R1 IHC-stained sections of tumor from a patient with a well-differentiated neuroendocrine tumor (patient in Fig. 2C) whose target lesions shrank 39% from baseline assessments. **B,** Distribution of CD200 H-scores and CD200R1-positive immune cells (% positive cells) at baseline. Pink data points present the CD200 H-score and CD200R1 %–positive immune cell value for the patient tumor shown in **A** [CD200 H-score of 155; CD200R1 %–positive immune cells of <1%]. **C,** Baseline CD200 tumor cell membrane staining intensity and frequency (%) for 19 patients with evaluable archival tumor tissue that received pharmacologic active doses of 23ME-00610 (≥60 mg) and sorted by PR, SD, or PD. ASPS, alveolar soft part sarcoma; CESC, cervical squamous cell carcinoma; ccSCA, clear cell chondrosarcoma; H&E, hematoxylin and eosin; LSA, leiomyosarcoma; NSCLC, non–small cell lung cancer; SKCM, cutaneous melanoma; THCA, papillary thyroid carcinoma.

The expression of CD200 and CD200R1 was evaluated by IHC in FFPE archival or baseline tumor biopsy samples collected from study participants (Fig. 3A; Supplementary Fig. S3A and S3B). Tumor tissue from 21 participants (75%) contained an adequate number of tumor cells to be evaluable for expression of CD200, and 12 of the 21 (57%) had detectable tumor cell CD200 expression (CD200 H-score > 0, assay range 0–300). The mean CD200 H-score for tumor cell CD200 expression was 25 [median (range): 1 (0–155); Fig. 3B]. Tumor tissue from 20 participants (71%) were evaluable for CD200R1 expression, and 19 of 20 (95%) had detectable immune cell expression of CD200R1. The mean percentage of immune cells expressing CD200R1 was 3.8% [median (range): 2.5% (0%–15%)], with expression observed on rare tumor cells [mean, median (range): 1.2%, 0% (0%–5%); Fig. 3B]. From baseline CD200 tumor cell membrane staining intensity and CD200R1 immune cell staining using validated assays, a potential trend between CD200 expression level (H-score) on tumor cells with clinical benefit (PR/SD versus PD) in participants treated with pharmacologic doses (≥60 mg) 23ME-00610 was observed (Fig. 3C; Supplementary Fig. S3C). The participant with a RECIST-confirmed PR (Fig. 2C) had detectable CD200 (with 100% tumor cell expression CD200) and the highest H-score (155) observed in evaluable participant data (Fig. 3A–C).

A total of 14 of 28 participants (i.e., 50%) who were genotyped by saliva-based testing were of European ancestry and had genotyping data available for calculation of immune-related and cancer PRSs (Supplementary Fig. S4). Because the sample size was small, differences in participant PRSs from a healthy reference population and correlative analyses with safety and efficacy were not conducted.

## Discussion

Although the development of immune checkpoint inhibitors (ICI) that enhance the immune system’s ability to identify and kill cancer cells has altered the treatment paradigm for a broad range of cancers (37), only a minority of cancer patients receive durable benefit from current immune-oncology (IO) therapies, pointing to an urgent need for the identification of additional immune checkpoints and novel biology to be addressed by new therapies. 23ME-00610 is a novel anti-CD200R1 mAb that antagonizes CD200-mediated immune suppression.

The safety and tolerability profile of 23ME-00610 was similar to the profile for other ICIs (31), with endocrine and cutaneous irAEs being the most commonly observed. Relative to other ICIs, the cutaneous irAEs observed with 23ME-00610 were generally responsive to antihistamine treatment (with one patient treated with oral and topical corticosteroids) and were maculopapular and sometimes urticarial in appearance, rather than the acneiform or eczema-like rashes typically seen with other ICIs (38). irAEs were observed at doses ≥60 mg, consistent with the PK/PD profile and predicted by exposure–effect modeling. Whereas the majority of irAEs occurred during the first cycle of 23ME-00610 treatment, a subset of irAEs had a delayed onset occurring after cycle 1, as seen previously with ICI treatment (31, 38, 39). irAEs were observed in three of five participants (60%) with clinical benefit (PR or SD > 6 months), including maculopapular rash for the participant with pancreatic neuroendocrine cancer with PR and a rash and thyroid-stimulating hormone increase in the patient with resolution of ascites, suggesting, at least numerically, that patients with clinical activity to 23ME-00610 may be more likely to have an irAE. For ICIs like 23ME-00610, irAEs are established to be associated with improved efficacy (39–45). Finally, the safety and tolerability profile for anti-CD200R1 23ME-00610 in the FIH participants on this study seems to largely overlap with the preliminary safety profile of the anti-CD200 antagonist samalizumab (46). Like with samalizumab, rash, pruritis, and urticaria were observed for 23ME-00610; however, samalizumab was primarily evaluated in hematologic malignancies rather than solid tumors, so the increased number of hematologic AEs seen with samalizumab and not with 23ME-00610 are more likely to be due to the underlying disease tested rather than a specific effect of CD200 versus CD200R1 antagonism.

23ME-00610 exhibited typical IgG1 antibody therapeutic PK characteristics, including a biphasic PK profile and approximately two-week half-life (by noncompartmental analysis). Doses ≥60 mg exhibited linear PK, moderate (∼2-fold) accumulation with Q3W dosing, and saturated circulating CD200R1. The PK/PD data from this study provide evidence for maximal CD200/R1 blockade with 23ME-00610, peripherally and potentially in the tumor. Whereas a cross-study comparison of PK/PD in different participant populations should be approached with caution, for example, due to differences in target expression and patient population, it should be noted that 23ME-00610 seems to antagonize the CD200/R1 pathway to a significantly greater degree than the anti-CD200 antibody samalizumab, which was unable to saturate CD200 at doses up to 500 mg/m^2^ (46).

For antagonist ICIs such as 23ME-00610, complete or near-complete target engagement is needed to elicit anticancer activity (47, 48). Although the PD data demonstrated durable blockade of soluble and membranous CD200R1 on immune cells from blood samples for participants dosed with 60 mg or more, this dose is unlikely to be sufficient to maximize pharmacology in tumor lesions; up to 10- to 20-fold higher doses of 23ME-00610 may be needed to saturate CD200R1 in the tumor microenvironment for the following reasons. Based on the published literature of other ICIs, only ∼10% of 23ME-00610 is expected to penetrate the tumor (48, 49). Additionally, tumor penetration has been shown to vary based on tumor type, disease burden, and anatomical location and vascularization of metastases, with ∼2-fold variation in drug penetration observed between lesions in the same patient (49). Furthermore, variability of tumor expression of CD200R1 among lesions and between tumor types, similar to PD-1, can result in an additional ∼2-fold difference in drug uptake based on target expression (50). Thus, a dose of 1,400 mg (∼20 × peripheral-to-tumor saturation ratio) increases the likelihood that the majority of patients will achieve sufficient drug levels all tumor lesions for complete target saturation beginning in cycle 1.

The primary objective of the phase 1 portion of this study was to identify the MTD and/or an initial dose to be tested in the phase 2a expansion phase. For 23ME-00610, the MTD was not reached. The PK target goal for this study was chosen to reduce the likelihood of subtherapeutic exposures in the expansion phase while maintaining tolerable dosing. Early safety and antitumor efficacy data support doses ≥600 mg as sufficiently active; repeated 600 mg Q3W doses achieved the PK target by cycle 2 for all evaluable participants. Nonetheless, 1,400 mg was well tolerated and the only dose level for which all participants achieved the PK target in cycle 1. The mean serum C_trough_ for 1,400 mg exceeded the projected efficacious PK target (i.e., >EC_90_ in the tumor), whereas the serum C_trough_ for two of five participants (40%) at the 600 mg level was below the EC_90_ target. Relative to doses ≤600 mg, 1,400 mg increases the likelihood that the majority of the patient population achieves the tumor EC_90_ PK target in cycle 1; thus, it was chosen as the dose to initiate preliminary efficacy assessments in the expansion phase. It is acknowledged that this dose escalation portion of the study is insufficient to identity the optimal biological dose of 23ME-00610. Additional 23ME-00610 dose levels (600 and 1,400 mg) are currently under investigation in cohorts of participants with ovarian cancers or neuroendocrine neoplasms (NCT05199272). These and future studies may support alternative doses and/or alternative dosing frequency, as well as identification of the optimal biologic dose of 23ME-00610.

Tumor shrinkage and durable treatment duration >12 months was observed for two participants in this study: one participant with esophageal adenocarcinoma, and a second participant with well-differentiated pancreatic neuroendocrine tumor. The participant with esophageal adenocarcinoma had prior nivolumab therapy, whereas the participant with neuroendocrine cancer was IO-naïve; both developed irAEs from 23ME-00610. The tumor cells of the neuroendocrine cancer of this latter patient had high expression of CD200 (H-score 155); unfortunately, CD200 could not be assessed in the archival tumor sample of the patient with esophageal adenocarcinoma due to insufficient tumor cells in the sample. A trend for clinical benefit among participants with high CD200 tumor expression was observed in a preliminary analysis of the phase 2a portion of this trial (51), particularly in neuroendocrine neoplasms (52) and renal cell carcinomas (53). Thus, further exploration of CD200 tumor expression as a potential predictive biomarker for 23ME-00610 clinical benefit is planned. In the current study, prior irAE data from prior IO therapies were not collected, and participants with a history of grade ≥3 immune-mediated toxicity considered related to prior immunotherapy were excluded; as a result, relationships between prior irAEs from other therapies and clinical benefit from 23ME-00610 were not conducted.

Germline PRSs have previously been evaluated post hoc in subsets of patients with cancer with genetic information who were treated with immune-oncology agents. These PRSs have demonstrated correlations with development of irAEs such as rashes and hypothyroidism and have also correlated in overall survival improvements for immune-oncology treatment, suggesting the potential utility of germline genetic scores to be used as predictive biomarker (26, 27, 54–56). In this study, prespecified, normalized PRSs could be calculated for each patient, and whereas the data were exploratory and expectedly inconclusive, the study demonstrated that real-time genotyping can be prospectively implemented in early-phase trials, and these analyses may be incorporated into clinical development planning for early assessment of potential biomarkers.

This FIH study for 23ME-00610 achieved well-tolerated doses that are likely to completely inhibit CD200R1 in the tumor. 23ME-00610 had a favorable PK/PD profile that supports Q3W dosing and a promising safety and tolerability profile that supports further study. Observed irAEs were consistent with 23ME-00610–mediated immune modulation and with the PK/PD data that demonstrated maximal peripheral saturation of CD200R1 on immune cells. Analysis of participant tumors suggested that patients with high tumor CD200 expression may be more likely to have clinical benefit with CD200R1 inhibition, which suggests promise as a predictive biomarker for efficacy. The dose escalation data supported a preliminary rcommended phase 2 dose of 1,400 mg for the expansion phase and additional dose optimization at 600 and 1,400 mg in populations that have showed preliminary evidence of clinical benefit.

## Supplementary Material

Supplementary Data Supplemental_all tables and figures

Figure S4 Supplemental Figure S4

Figure S3 Supplemental Figure S3

Figure S2 Supplemental Figure S2

Figure S1 Supplemental Figure S1

## References

[1] Rygiel TP , Meyaard L. CD200R signaling in tumor tolerance and inflammation: a tricky balance. Curr Opin Immunol 2012;24:233–8. 22264927 10.1016/j.coi.2012.01.002

[2] Mihrshahi R , Barclay AN, Brown MH. Essential roles for Dok2 and RasGAP in CD200 receptor-mediated regulation of human myeloid cells. J Immunol 2009;183:4879–86. 19786546 10.4049/jimmunol.0901531 PMC2788151

[3] Zhang S , Cherwinski H, Sedgwick JD, Phillips JH. Molecular mechanisms of CD200 inhibition of mast cell activation. J Immunol 2004;173:6786–93. 15557172 10.4049/jimmunol.173.11.6786

[4] Misstear K , Chanas SA, Rezaee SAR, Colman R, Quinn LL, Long HM, . Suppression of antigen-specific T cell responses by the Kaposi’s sarcoma-associated herpesvirus viral OX2 protein and its cellular orthologue, CD200. J Virol 2012;86:6246–57. 22491458 10.1128/JVI.07168-11 PMC3372194

[5] Herbrich S , Baran N, Cai T, Weng C, Aitken MJL, Post SM, . Overexpression of CD200 is a stem cell-specific mechanism of immune evasion in AML. J Immunother Cancer 2021;9:e002968. 34326171 10.1136/jitc-2021-002968 PMC8323398

[6] Shafiei-Jahani P , Helou DG, Hurrell BP, Howard E, Quach C, Painter JD, . CD200–CD200R immune checkpoint engagement regulates ILC2 effector function and ameliorates lung inflammation in asthma. Nat Commun 2021;12:2526. 33953190 10.1038/s41467-021-22832-7 PMC8100131

[7] Choe D , Choi D. Cancel cancer: the immunotherapeutic potential of CD200/CD200R blockade. Front Oncol 2023;13:1088038. 36756156 10.3389/fonc.2023.1088038 PMC9900175

[8] Wright GJ , Cherwinski H, Foster-Cuevas M, Brooke G, Puklavec MJ, Bigler M, . Characterization of the CD200 receptor family in mice and humans and their interactions with CD200. J Immunol 2003;171:3034–46. 12960329 10.4049/jimmunol.171.6.3034

[9] Rijkers ESK , de Ruiter T, Baridi A, Veninga H, Hoek RM, Meyaard L. The inhibitory CD200R is differentially expressed on human and mouse T and B lymphocytes. Mol Immunol 2008;45:1126–35. 17714785 10.1016/j.molimm.2007.07.013

[10] Fenaux J , Fang X, Huang Y-M, Melero C, Bonnans C, Lowe EL, . 23ME-00610, a genetically informed, first-in-class antibody targeting CD200R1 to enhance antitumor T cell function. Oncoimmunology 2023;12:2217737. 37288324 10.1080/2162402X.2023.2217737 PMC10243377

[11] Khan M , Arooj S, Wang H. NK cell-based immune checkpoint inhibition. Front Immunol 2020;11:167. 32117298 10.3389/fimmu.2020.00167 PMC7031489

[12] Fang X , Chung W-J, Fenaux J, Chen A, Batada N, Schmidt M, . Abstract 603: Discovery of CD200R1 as a novel immuno-oncology target using pleiotropic signals from 23andMe’s genetic and health survey database. Cancer Res 2022;82(Suppl 12):603.

[13] Wright GJ , Puklavec MJ, Willis AC, Hoek RM, Sedgwick JD, Brown MH, . Lymphoid/neuronal cell surface OX2 glycoprotein recognizes a novel receptor on macrophages implicated in the control of their function. Immunity 2000;13:233–42. 10981966 10.1016/s1074-7613(00)00023-6

[14] Wright GJ , Jones M, Puklavec MJ, Brown MH, Barclay AN. The unusual distribution of the neuronal/lymphoid cell surface CD200 (OX2) glycoprotein is conserved in humans. Immunology 2001;102:173–9. 11260322 10.1046/j.1365-2567.2001.01163.x PMC1783166

[15] Moreaux J , Hose D, Reme T, Jourdan E, Hundemer M, Legouffe E, . CD200 is a new prognostic factor in multiple myeloma. Blood 2006;108:4194–7. 16946299 10.1182/blood-2006-06-029355

[16] Tonks A , Hills R, White P, Rosie B, Mills KI, Burnett AK, . CD200 as a prognostic factor in acute myeloid leukaemia. Leukemia 2007;21:566–8. 17252007 10.1038/sj.leu.2404559

[17] Kumar MR , Duan S, Yadav S, Jarret A, Palumbo T, Yan Z, . New insights into targeting the CD200R1 pathway in T and NK cells using 23me-00610 as a single agent or in combination [abstract]. In: Proceedings of the American Association for Cancer Research Annual Meeting 2024; Part 1 (Regular Abstracts); 2024 Apr 5–10; San Diego, CA. Philadelphia (PA): AACR; 2025.

[18] Wei SC , Anang N-AAS, Sharma R, Andrews MC, Reuben A, Levine JH, . Combination anti–CTLA-4 plus anti–PD-1 checkpoint blockade utilizes cellular mechanisms partially distinct from monotherapies. Proc Natl Acad Sci U S A 2019;116:22699–709. 31636208 10.1073/pnas.1821218116 PMC6842624

[19] Dai T , Sun H, Liban T, Vicente-Suarez I, Zhang B, Song Y, . A novel anti-LAG-3/TIGIT bispecific antibody exhibits potent anti-tumor efficacy in mouse models as monotherapy or in combination with PD-1 antibody. Sci Rep 2024;14:10661. 38724599 10.1038/s41598-024-61477-6 PMC11082181

[20] Kummar S , Razak AA, Laurie S, Kell S, Glatt D, Majeed SR, . Abstract CT174: First-in-class anti-CD200R1 antibody 23ME-00610 in patients with advanced solid malignancies: phase 1 results. Cancer Res 2023;83(Suppl 8):CT174.

[21] Glatt DM , Kell S, Schmidt M, Majeed SR. 609 Phase 1/2a dose selection of 23ME-00610, a first-in-class anti-CD200R1 antibody, in patients with advanced solid malignancies. J Immunother Cancer 2023;11(Suppl 1):693.

[22] Melero C , Budiardjo SJ, Daruwalla A, Larrabee L, Ganichkin O, Heiler AJ, . CD200R1 immune checkpoint blockade by the first-in-human anti-CD200R1 antibody 23ME-00610: molecular mechanism and engineering of a surrogate antibody. MAbs 2024;16:2410316. 39402718 10.1080/19420862.2024.2410316 PMC11485749

[23] Garon EB , Rizvi NA, Hui R, Leighl N, Balmanoukian AS, Eder JP, . KEYNOTE-001 Investigators; Pembrolizumab for the treatment of non-small-cell lung cancer. N Engl J Med 2015;372:2018–28. 25891174 10.1056/NEJMoa1501824

[24] Borghaei H , Paz-Ares L, Horn L, Spigel DR, Steins M, Ready NE, . Nivolumab versus docetaxel in advanced nonsquamous non–small-cell lung cancer. N Engl J Med 2015;373:1627–39. 26412456 10.1056/NEJMoa1507643 PMC5705936

[25] Rittmeyer A , Barlesi F, Waterkamp D, Park K, Ciardiello F, von Pawel J, . Atezolizumab versus docetaxel in patients with previously treated non-small-cell lung cancer (OAK): a phase 3, open-label, multicentre randomised controlled trial. Lancet 2017;389:255–65. 27979383 10.1016/S0140-6736(16)32517-X PMC6886121

[26] Khan Z , Di Nucci F, Kwan A, Hammer C, Mariathasan S, Rouilly V, . Polygenic risk for skin autoimmunity impacts immune checkpoint blockade in bladder cancer. Proc Natl Acad Sci U S A 2020;117:12288–94. 32430334 10.1073/pnas.1922867117 PMC7275757

[27] Khan Z , Hammer C, Carroll J, Di Nucci F, Acosta SL, Maiya V, . Genetic variation associated with thyroid autoimmunity shapes the systemic immune response to PD-1 checkpoint blockade. Nat Commun 2021;12:3355. 34099659 10.1038/s41467-021-23661-4 PMC8184890

[28] Middha P , Thummalapalli R, Betti MJ, Yao L, Quandt Z, Balaratnam K, . Polygenic risk score for ulcerative colitis predicts immune checkpoint inhibitor-mediated colitis. Nat Commun 2024;15:2568. 38531883 10.1038/s41467-023-44512-4 PMC10966072

[29] Lambert SA , Gil L, Jupp S, Ritchie SC, Xu Y, Buniello A, . The Polygenic Score Catalog as an open database for reproducibility and systematic evaluation. Nat Genet 2021;53:420–5. 33692568 10.1038/s41588-021-00783-5 PMC11165303

[30] Hendrikx JJMA , Haanen JBAG, Voest EE, Schellens JHM, Huitema ADR, Beijnen JH. Fixed dosing of monoclonal antibodies in oncology. Oncologist Oct 2017;22:1212–21. 28754722 10.1634/theoncologist.2017-0167 PMC5634778

[31] Ramos-Casals M , Brahmer JR, Callahan MK, Flores-Chávez A, Keegan N, Khamashta MA, . Immune-related adverse events of checkpoint inhibitors. Nat Rev Dis Primers 2020;6:38. 32382051 10.1038/s41572-020-0160-6 PMC9728094

[32] Brahmer JR , Lacchetti C, Schneider BJ, Atkins MB, Brassil KJ, Caterino JM, . Management of immune-related adverse events in patients treated with immune checkpoint inhibitor therapy: American Society of Clinical Oncology Clinical Practice guideline. J Clin Oncol 2018;36:1714–68. 29442540 10.1200/JCO.2017.77.6385 PMC6481621

[33] Eisenhauer EA , Therasse P, Bogaerts J, Schwartz LH, Sargent D, Ford R, . New response evaluation criteria in solid tumours: revised RECIST guideline (version 1.1). Eur J Cancer 2009;45:228–47. 19097774 10.1016/j.ejca.2008.10.026

[34] Seymour L , Bogaerts J, Perrone A, Ford R, Schwartz LH, Mandrekar S, . iRECIST: guidelines for response criteria for use in trials testing immunotherapeutics. Lancet Oncol 2017;18:e143–52. 28271869 10.1016/S1470-2045(17)30074-8 PMC5648544

[35] McCarthy S , Das S, Kretzschmar W, Delaneau O, Wood AR, Teumer A, . A reference panel of 64,976 haplotypes for genotype imputation. Nat Genet 2016;48:1279–83. 27548312 10.1038/ng.3643 PMC5388176

[36] Tung JY , Do CB, Hinds DA, Kiefer AK, Macpherson JM, Chowdry AB, . Efficient replication of over 180 genetic associations with self-reported medical data. PLoS One 2011;6:e23473. 21858135 10.1371/journal.pone.0023473 PMC3157390

[37] National Comprehensive Cancer Network [NCCN] . Guidances and clinical resources. [cited 2023 Oct 11]. Available from: https://www.nccn.org/professionals/physician_gls/default.aspx#site.

[38] Sibaud V . Dermatologic reactions to immune checkpoint inhibitors: skin toxicities and immunotherapy. Am J Clin Dermatol 2018;19:345–61. 29256113 10.1007/s40257-017-0336-3

[39] Socinski MA , Jotte RM, Cappuzzo F, Nishio M, Mok TSK, Reck M, . Association of immune-related adverse events with efficacy of atezolizumab in patients with non-small cell lung cancer: pooled analyses of the phase 3 IMpower130, IMpower132, and IMpower150 randomized clinical trials. JAMA Oncol 2023;9:527–35. 36795388 10.1001/jamaoncol.2022.7711 PMC9936386

[40] Akamatsu H , Murakami E, Oyanagi J, Shibaki R, Kaki T, Takase E, . Immune-related adverse events by immune checkpoint inhibitors significantly predict durable efficacy even in responders with advanced non-small cell lung cancer. Oncologist 2020;25:e679–83. 32297443 10.1634/theoncologist.2019-0299 PMC7160399

[41] Hussaini S , Chehade R, Boldt RG, Raphael J, Blanchette P, Maleki Vareki S, . Association between immune-related side effects and efficacy and benefit of immune checkpoint inhibitors—a systematic review and meta-analysis. Cancer Treat Rev 2021;92:102134. 33302134 10.1016/j.ctrv.2020.102134

[42] Remon J , Reguart N, Auclin E, Besse B. Immune-related adverse events and outcomes in patients with advanced non-small cell lung cancer: a predictive marker of efficacy? J Thorac Oncol 2019;14:963–7. 31027971 10.1016/j.jtho.2019.02.031

[43] Cortellini A , Chiari R, Ricciuti B, Metro G, Perrone F, Tiseo M, . Correlations between the immune-related adverse events spectrum and efficacy of anti-PD1 immunotherapy in NSCLC patients. Clin Lung Cancer 2019;20:237–47.e1. 30885550 10.1016/j.cllc.2019.02.006

[44] Luo J , Martucci VL, Quandt Z, Groha S, Murray MH, Lovly CM, . Immunotherapy-mediated thyroid dysfunction: genetic risk and impact on outcomes with PD-1 blockade in non-small cell lung cancer. Clin Cancer Res 2021;27:5131–40. 34244291 10.1158/1078-0432.CCR-21-0921 PMC8815444

[45] Eggermont AMM , Kicinski M, Blank CU, Mandala M, Long GV, Atkinson V, . Association between immune-related adverse events and recurrence-free survival among patients with stage III melanoma randomized to receive pembrolizumab or placebo: a secondary analysis of a randomized clinical trial. JAMA Oncol 2020;6:519–27. 31895407 10.1001/jamaoncol.2019.5570 PMC6990933

[46] Mahadevan D , Lanasa MC, Farber C, Pandey M, Whelden M, Faas SJ, . Phase I study of samalizumab in chronic lymphocytic leukemia and multiple myeloma: blockade of the immune checkpoint CD200. J Immunother Cancer 2019;7:227. 31443741 10.1186/s40425-019-0710-1 PMC6708181

[47] Patnaik A , Kang SP, Rasco D, Papadopoulos KP, Elassaiss-Schaap J, Beeram M, . Phase I study of pembrolizumab (MK-3475; anti-PD-1 monoclonal antibody) in patients with advanced solid tumors. Clin Cancer Res 2015;21:4286–93. 25977344 10.1158/1078-0432.CCR-14-2607

[48] Li TR , Chatterjee M, Lala M, Abraham AK, Freshwater T, Jain L, . Pivotal dose of pembrolizumab: a dose-finding strategy for immuno-oncology. Clin Pharmacol Ther 2021;110:200–9. 33462831 10.1002/cpt.2170

[49] Bensch F , van der Veen EL, Lub-de Hooge MN, Jorritsma-Smit A, Boellaard R, Kok IC, . ^89^Zr-atezolizumab imaging as a non-invasive approach to assess clinical response to PD-L1 blockade in cancer. Nat Med 2018;24:1852–8. 30478423 10.1038/s41591-018-0255-8

[50] Niemeijer AN , Leung D, Huisman MC, Bahce I, Hoekstra OS, van Dongen GAMS, . Whole body PD-1 and PD-L1 positron emission tomography in patients with non-small-cell lung cancer. Nat Commun 2018;9:4664. 30405135 10.1038/s41467-018-07131-y PMC6220188

[51] Razak AA , Naing A, Diep A, Hwang C, Reisetter A, Fuller Z, . 150P Phase I/IIa trial of CD200R1 inhibitor 23ME-00610: exploratory analyses of tissue-based and genetic biomarkers. Ann Oncol 2024;35(Suppl 2):S274–5.

[52] Rasco DW , Abdul Razak AR, Khaki AR, Spira AI, Hwang CC, Diep AN, . Safety, efficacy, and PKPD of 23ME-00610, a first-in-class anti-CD200R1 antibody, in patients with advanced neuroendocrine cancers: results from a multi-center multi-country phase 1/2a expansion cohort. J Clin Oncol 2024;42(Suppl 16):4129.

[53] Krystal J , Khaki AR, Spira AI, Abdul Razak AR, Maslyar D, Glatt D, . 1706P efficacy, safety and PKPD of 23ME-00610, a first-in-class anti-CD200R1 antibody, in patients with advanced or metastatic clear-cell renal cell carcinoma (ccRCC): results from a multi-center multi-country phase I/IIa expansion cohort. Ann Oncol 2024;35:S1023.

[54] Chat V , Ferguson R, Kirchhoff T. Germline genetic host factors as predictive biomarkers in immuno-oncology. Immunooncol Technol 2019;2:14–21. 35756849 10.1016/j.iotech.2019.08.001 PMC9216465

[55] Orrù V , Steri M, Sole G, Sidore C, Virdis F, Dei M, . Genetic variants regulating immune cell levels in health and disease. Cell 2013;155:242–56. 24074872 10.1016/j.cell.2013.08.041 PMC5541764

[56] Heilbron K , Mozaffari SV, Vacic V, Yue P, Wang W, Shi J, . Advancing drug discovery using the power of the human genome. J Pathol 2021;254:418–29. 33748968 10.1002/path.5664 PMC8251523

