# Supplementary material for: First-in-Human Study of 23ME-00610, an Antagonistic Antibody for Genetically Validated CD200R1 Immune Checkpoint, in Participants with Advanced Solid Malignancies
Source: Cancer Res Commun. 2025 Jan 15;5(1):94–105. doi: 10.1158/2767-9764.CRC-24-0568 (PMC11734590; doi:10.1158/2767-9764.CRC-24-0568)
Supplement: Supplementary Data — Supplemental_all tables and figures [file crc-24-0568_supplementary_data_suppsd1.docx]

**Supplemental Tables and Figures**

| **Supplemental Table S1** | Representativeness of Study Participants |
| --- | --- |
| **Supplemental Table S2** | Summary of Participant Disposition |
| **Supplemental Table S3** | Treatment Emergent Serious Adverse Events (TESAEs) |
| **Supplemental Table S4** | Summary Prevalence and Incidence of 23ME-00610 ADA |
| **Supplemental Table S5** | Best Overall Response by Dose Level per RECIST V1.1 |
| **Supplemental Table S6** | Duration of 23ME-00610 Exposure by Best Overall Response |
| **Supplemental Figure S1** | Study Design for 23ME-00610 First-in-Human Clinical Trial |
| **Supplemental Figure S2** | Scans from Screening to Week 24 for Participant with Esophageal Adenocarcinoma |
| **Supplemental Figure S3** | CD200 and CD200R1 staining intensity and distribution by clinical benefit |
| **Supplemental Figure S4** | Exploratory Germline Genetic Analysis of European-ancestry First-in-Human Trial Participants Treated with 23ME-00610 |

**Supplemental Table S1: Representativeness of Study Participants**

| **Cancer type(s) /subtype(s)/ stage(s)/ condition** | Enrolled participants had a histologically diagnosed locally advanced (unresectable) or metastatic solid cancer that had progressed after all available standard therapy for the specific tumor type or no further standard therapy existed. Primary tumor site for study participants included colorectal, pancreatic, neuroendocrine, esophageal, melanoma, sarcoma, breast, osteosarcoma, prostate, and endometrial, among others, and all were late stage (ie, Stage 3 or 4). |
| --- | --- |
| **Considerations related to:** | |
| **Sex** | Sex can play a role in cancer incidence, prognosis and mortality, with a 1 in 2 chance for men in developing some form of cancer in their lifetime, on average, and 1 in 3 chance for women, also on average. This study enrolled an equal number of males and females: 14 each. |
| **Age** | The median age for cancer diagnosis is between 60 and 70, and cancer risk rises more steeply from the age of 50-60. Eligible participants for this first-in-human study were at least 18 years of age. The median age of enrolled participants with advanced solid cancer for this study was 62. The youngest participant was 21, and the oldest participant was 80. |
| **Race/ethnicity** | In general, cancer incidence rates appear to be highest among White and Black/African American individuals, relative to Asian and Pacific Islanders, American Indian/Alaska Native, and Hispanic and Latino individuals. Recent studies analyzing race/ethnicity for Phase 1 clinical trials in North America have shown that White/Caucasian participants dominate, accounting for ~80-90% of the participant population. Black/African American, Asian, and Hispanic/Latino account for ~3-10%, and American Indian/Alaska Native are typically < 1%. With ~79% White, 4% Black/African American, 7% Asian, and 4% American Indian/Alaska Native. and approximately 22% that identified as Hispanic or Latino, this trial population was similar or outperformed historic benchmarks for diversity. |
| **Geography** | Cancer incidence rates in Canada and the US are similar, and both countries are among the top 10 in the word for age-standardized cancer incidence. This trial enrolled 82% from the US and 18% from Canada, largely driven by the number of sites initiated in each country and the timing of first patient enrolled for each site. |
| **Other considerations** | Typical for first-in-human criteria for preliminary evaluation of safety and efficacy, eligible participants had an Eastern Cooperative Oncology Group (ECOG) performance status of 0 or 1 and RECIST measurable or evaluable disease. As expected for a potential immune-modulatory biologic (mAb) like 23ME-00610 in participants with cancer, key exclusion criteria included presence of active autoimmune disease that required immunosuppressive treatment in the last 2 years, history of Grade ≥ 3 immune-mediated toxicity related to prior immunotherapy that led to discontinuation, or uncontrolled or symptomatic central nervous system metastases and or carcinomatous meningitis. |
| **Overall representativeness of this study** | The age, sex, and race/ethnicity distribution of our study is as expected for the cancer types enrolled and geography. Given the small sample size of 28 total participants, the study reasonably represents the cancer population to be expected for an advanced solid malignancies population in the US and Canada that had progressed after all available standard therapy for the specific tumor type or no further standard therapy existed. |

**Supplemental Table S2. Summary of Participant Disposition**

|  | **2 mg**  **(N=1)** | **6 mg**  **(N=1)** | **20 mg**  **(N=3)** | **60 mg**  **(N=4)** | **200 mg**  **(N=3)** | **600 mg**  **(N=8)** | **1400 mg**  **(N=8)** | **Total**  **(N=28)** |
| --- | --- | --- | --- | --- | --- | --- | --- | --- |
| **Participant Status** | | | | | | | | |
| On Treatment  (N, [%]) | 0 (0.0%) | 0 (0.0%) | 0 (0.0%) | 0 (0.0%) | 0 (0.0%) | 1 (33.3%) | 0 (0.0%) | 1 (3.6%) |
| Discontinued Treatment  (N, [%]) | 1 (100%) | 1 (100%) | 3 (100%) | 4 (100%) | 3 (100%) | 7 (87.5%) | 8 (100%) | 27 (96.4%) |
| Discontinued due to Disease Progression  (N, [%]) | 1 (100%) | 1 (100%) | 3 (100%) | 4 (100%) | 3 (100%) | 6 (75.0%) | 8 (100%) | 26 (92.9%) |
| Discontinued due to Adverse Event  (N, [%]) | 0 (0.0%) | 0 (0.0%) | 0 (0.0%) | 0 (0.0%) | 0 (0.0%) | 1 (12.5%) | 0 (0.0%) | 1 (3.6%) |

Data as of October 2, 2024.

**Supplemental Table S3: Treatment Emergent Serious Adverse Events (TESAEs)**

|  | **2 mg**  **(N=1)** | **6 mg (N=1)** | **20 mg (N=3)** | **60 mg (N=4)** | **200 mg (N=3)** | **600 mg (N=8)** | **1400 mg (N=8)** | **Total**  **(N=28)** |
| --- | --- | --- | --- | --- | --- | --- | --- | --- |
| Any TESAEs | 0 | 0 | 1 (33.3%) | 0 | 2 (66.7%) | 2 (25.0%) | 1 (12.5%) | 6 (21.4%) |
| Constipation | 0 | 0 | 1 (33.3%) | 0 | 0 | 0 | 0 | 1 (3.6%) |
| GI hemorrhage | 0 | 0 | 0 | 0 | 1 (33.3%) | 0 | 0 | 1 (3.6%) |
| Intestinal Obstruction | 0 | 0 | 0 | 0 | 0 | 1 (12.5%) | 0 | 1 (3.6%) |
| Lower GI hemorrhage | 0 | 0 | 1 (33.3%) | 0 | 0 | 0 | 0 | 1 (3.6%) |
| Cellulitis | 0 | 0 | 0 | 0 | 0 | 1 (12.5%) | 0 | 1 (3.6%) |
| Influenza | 0 | 0 | 0 | 0 | 1 (33.3%) | 0 | 0 | 1 (3.6%) |
| Pleural Effusion | 0 | 0 | 0 | 0 | 0 | 0 | 1 (12.5%) | 1 (3.6%) |

Data as of October 2, 2024.

Abbreviations: GI, Gastrointestinal.

**Supplemental Table S4: Summary Prevalence and Incidence of 23ME-00610 ADA**

| **ADA+, n (%)** | | | | | |
| --- | --- | --- | --- | --- | --- |
| **Dose**  **(mg)** | **N** | **ADA Prevalence at Baseline** | **ADA incidence (emergence)** | **Treatment-induced ADA** | **Treatment-boosted ADA** |
| 2 | 1 | 0 | 0 | 0 | 0 |
| 6 | 1 | 1 (100%) | 0 | 0 | 0 |
| 20 | 3 | 2 (67%) | 0 | 0 | 0 |
| 60 | 4 | 0 | 0 | 0 | 0 |
| 200 | 3 | 0 | 0 | 0 | 0 |
| 600 | 6 | 0 | 0 | 0 | 0 |
| 1400 | 5 | 0 | 0 | 0 | 0 |
| Total | 23 | 3 (13.6%) | 0 | 0 | 0 |

Note: % = 100 x n/N. Five (5) patients were missing either a baseline or post-baseline assessment; thus, N=23 in the ADA/immunogenicity population. ADA incidence (emergence) is defined as sum of treatment-induced and treatment-boosted ADA. Treatment-Induced ADA is defined as ADA negative at baseline and positive at any post-Baseline. Treatment-Boosted ADA is defined as ADA positive at baseline and positive post-baseline with a titer that is at least 4-fold higher post-baseline compared to baseline.

Abbreviations: ADA; Anti-drug antibodies.

**Supplemental Table S5: Best Overall Response by Dose Level per RECIST V1.1**

|  | **23ME-00610 Dose Level and N** | | | | | |
| --- | --- | --- | --- | --- | --- | --- |
| **Best Overall Response** | **1400 mg**  **(N=8)** | **600 mg**  **(N=8)** | **≥ 600 mg**  **(N=16)** | **≥ 200 mg**  **(N=19)^^^** | **≥ 60 mg**  **(N=23)^%^** | **≥ 2 mg**  **(N=28)** |
| Complete Response (CR) | 0 | 0 | 0 | 0 | 0 | 0 (0.0%) |
| Partial Response (PR) | 0 | 0 | 0 | 1 (5.3%) | 1 (4.3%) | 1 (3.6%) |
| Stable Disease (SD) | 3 (37.5%) | 4 (50%) | 7 (43.8%) | 8 (42.1%) | 10 (43.5%) | 13 (46.4%) |
| Progressive Disease (PD) | 3 (37.5%) | 4 (50%) | 7 (43.8%) | 8 (42.1%) | 10 (4.5%) | 12 (42.9%) |
| Not Evaluable (NE)* | 2 (25%) | 0 | 2 (12.5%) | 2 (10.5%) | 2 (8.7%) | 2 (7.1%) |

Data as of October 2, 2024.

* post-baseline tumor assessments were not performed

^ Three participants enrolled at 200 mg had their 23ME-00610 dose escalated to 600 mg; 1 of the 3 (33%) had a partial response, and 2 of the 3 (67%) had stable disease.

% One participant enrolled at 60 mg had their 23ME-00610 dose escalated to 200 mg.

**Supplemental Table S6: Duration of 23ME-00610 Exposure by Best Overall Response**

|  |  | **Duration of 23ME-00610 Exposure (days)** | | |
| --- | --- | --- | --- | --- |
| **Best Overall Response per RECIST v1.1** | **N** | **Median** | **Minimum** | **Maximum** |
| All participants | 28 | 93.5 | 1 | 690 |
| Partial Response and/or Stable Disease | 14 | 211 | 23 | 690 |

Data as of October 2, 2024.

**Supplemental Figure S1: Study Design for 23ME-00610 First-in-Human Clinical Trial**

**Supplemental Figure S1.** The Phase 1/2a first-in-human (FIH) multicenter study of 23ME-00610 utilized an accelerated titration followed by "3+3" open-label design. 23ME-00610 was administered by IV infusion every 3 weeks (Q3W) in participants with histologically diagnosed locally advanced (unresectable) or metastatic solid cancer that had progressed after all available standard therapy for the specific tumor type or no further standard therapy existed. The study design included a PK/PD Backfill cohort (N ≤ 12 participants) at levels anticipated to be in the pharmacologically active dose range (ie, ≥ 600 mg).

Abbreviations: IV, intravenously; PK/PD, pharmacokinetics/pharmacodynamics.

**Supplemental Figure S2. Scans from Screening to Week 24 for Participant with Esophageal Adenocarcinoma**


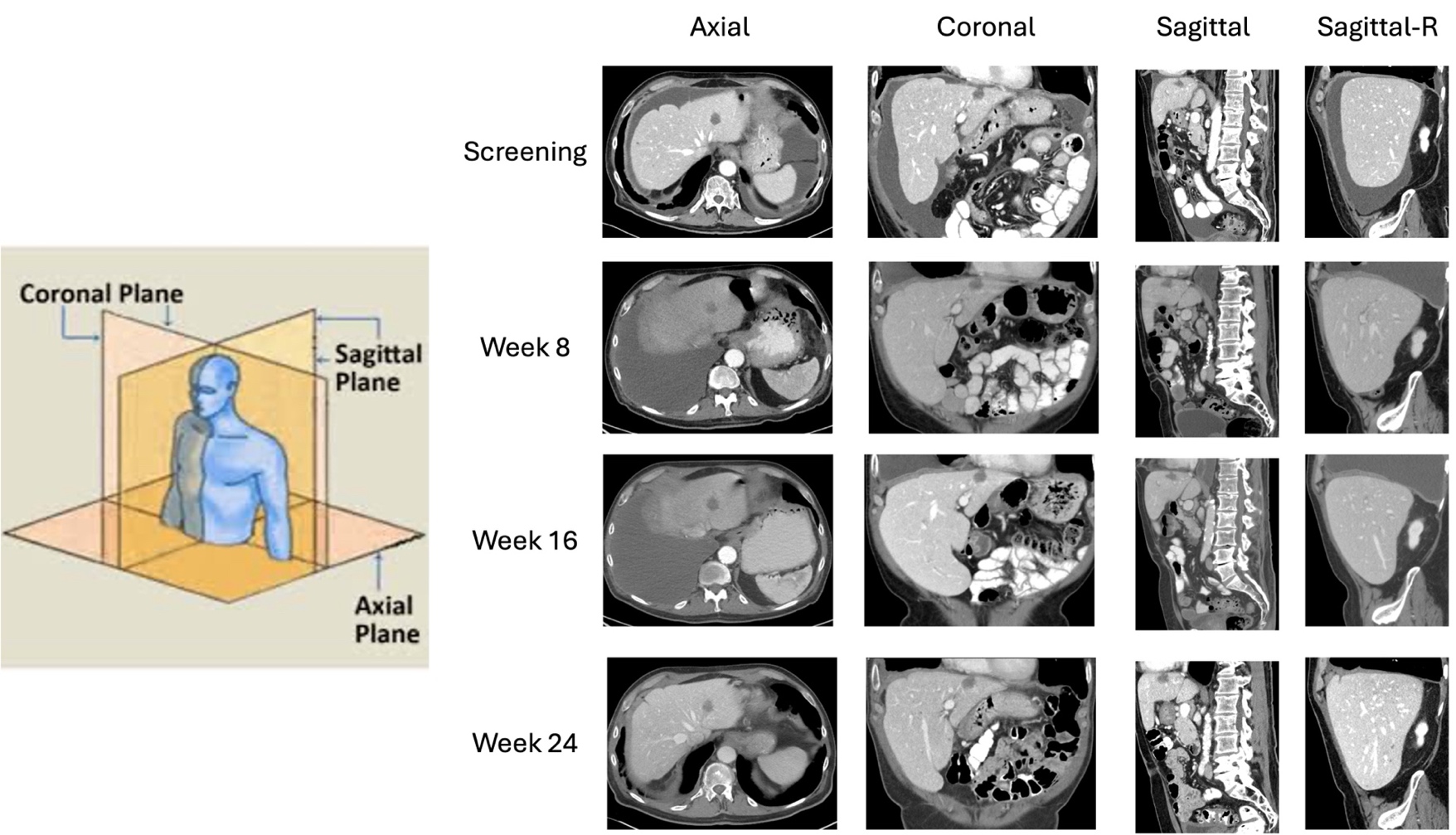


**Supplemental Figure S2.** 66-year old male with esophageal cancer resected in 2019 with recurrent metastatic disease in 2022. Following progression on combination therapy with FOLFOX-nivolumab (FOLFOX = folinic acid, 5-fluorouracil, and oxaliplatin), the patient enrolled on the 23ME-00610 Phase 1 study at the dose escalation dose level of 600 mg administered once every three weeks (Q3W). At baseline (Screening), the patient had liver metastases and ascites. After the first dose, the patient developed an immune related adverse event of rash. Concurrently, the ascites resolved; however, massive right sided pleural effusion developed. With continued 23ME-00610 study treatment, the pleural effusion resolved concurrently with the event of immune related hypothyroidism by Week 24. The patient remains with improved clinical status and without progression of disease after initiating study treatment (> 21 months as of the October 2, 2024 data cutoff). Due to insufficient tumor cell content in the archival tumor sample, tumor CD200 expression was unable to be determined.

**Supplemental Figure S3. CD200 and CD200R1 staining intensity and distribution by clinical benefit**

**S3A)
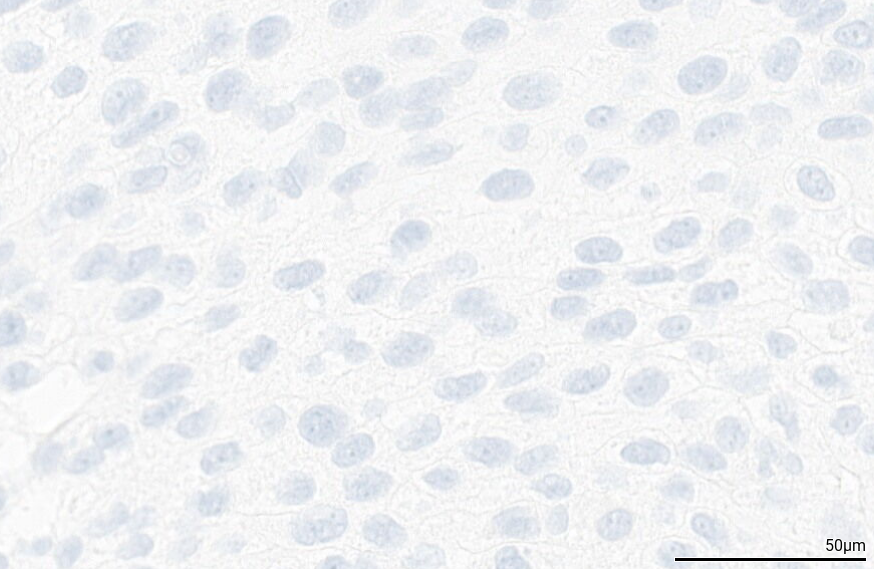
**

**
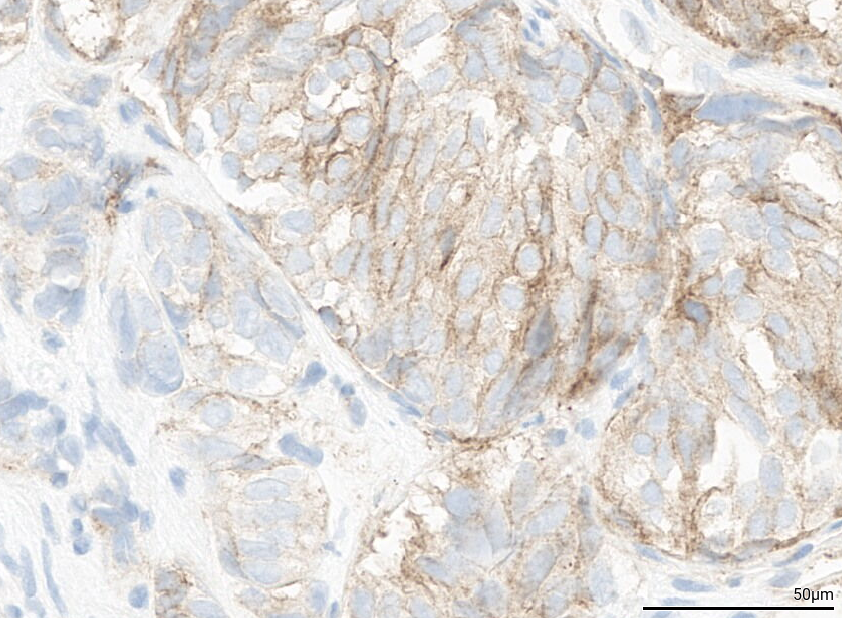
**

**0**

**+1**

**
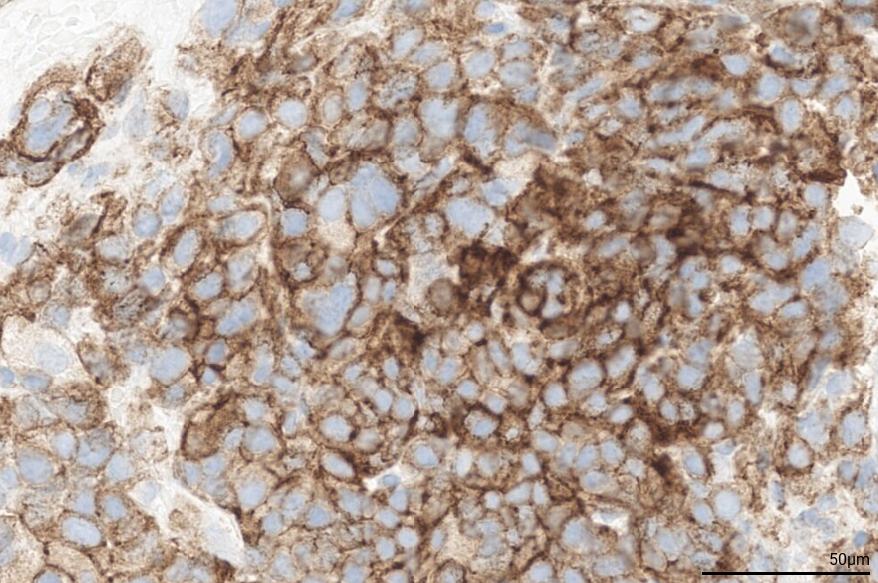

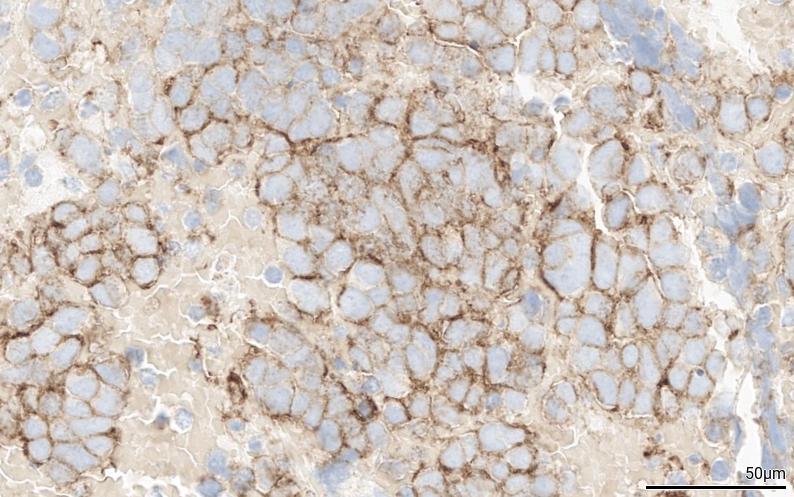
**

**+3**

**+2**

**Supplemental Figure 3A:** Representative images of tumors with no CD200 membranous expression (0), l

**S3B)**

**
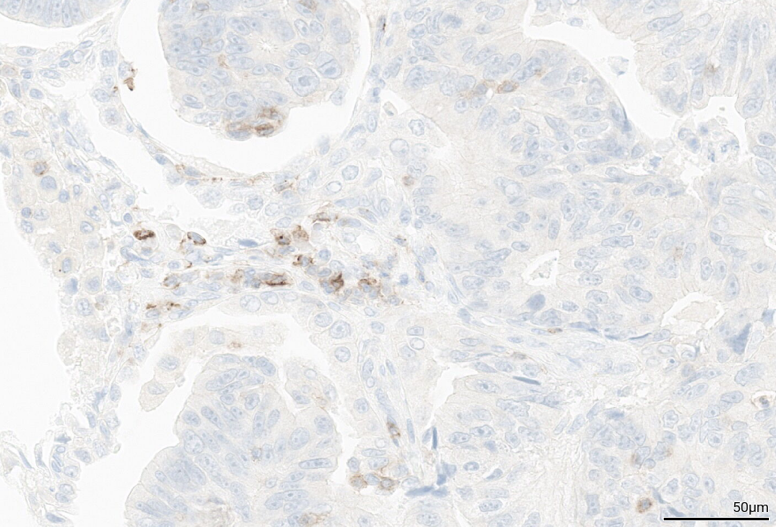

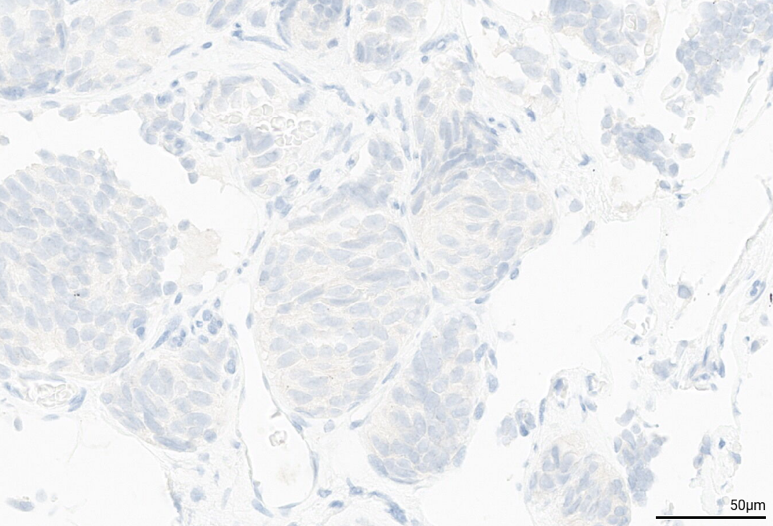
**

**3%**

**0%**

**
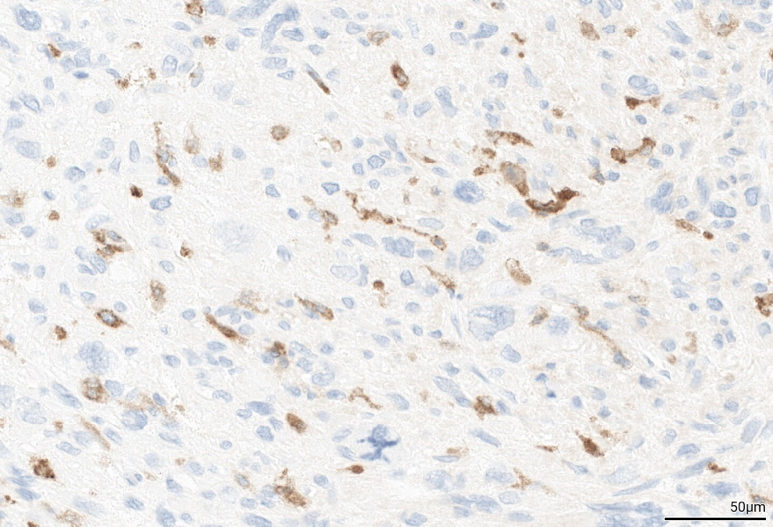

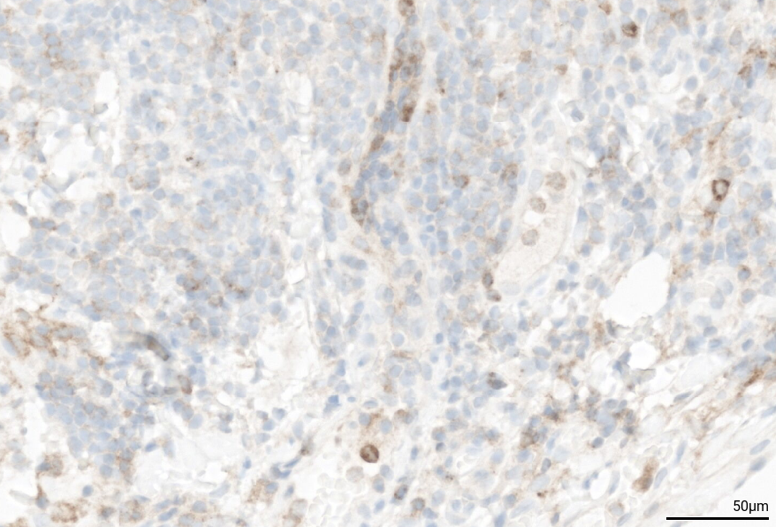
**

**15%**

**8%**

**S3C)**


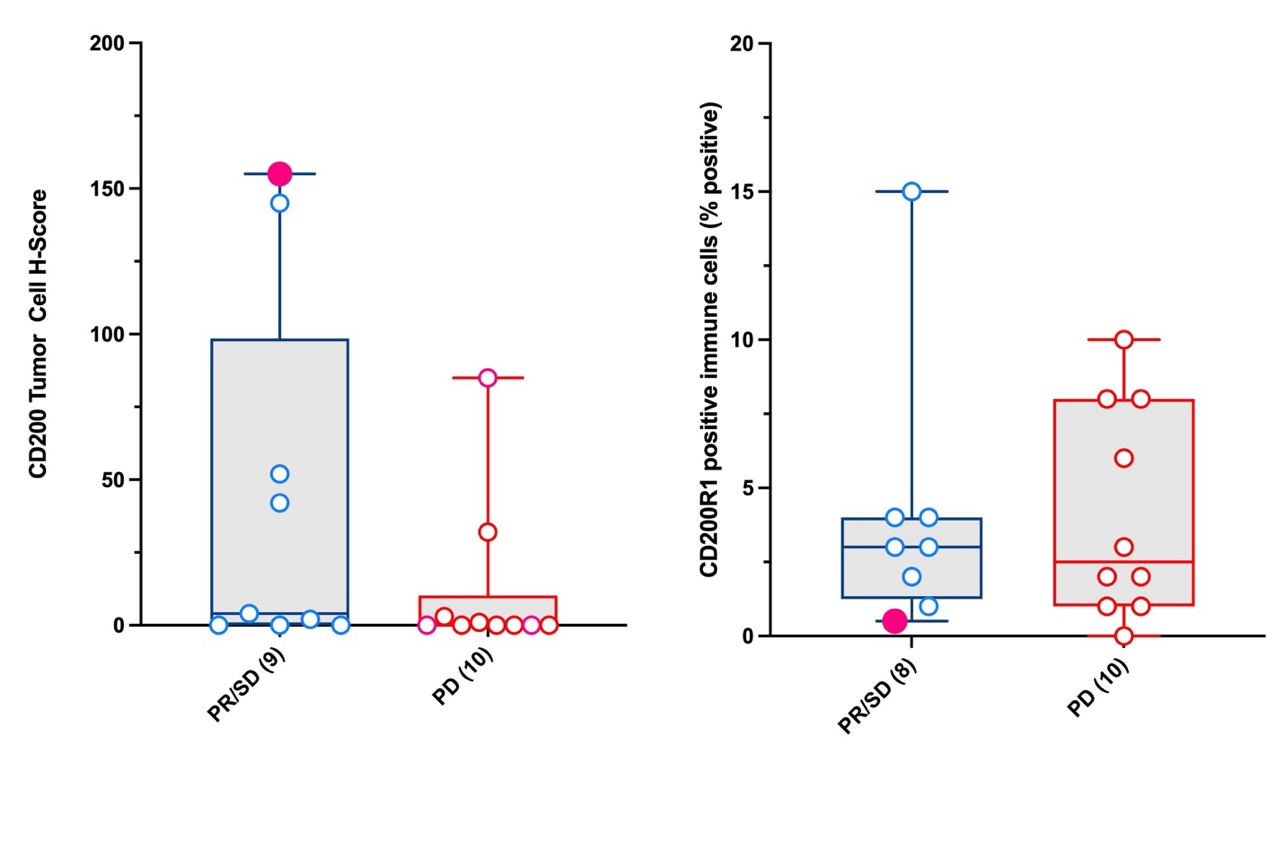


**Supplemental Figure S3.** Representative images of tumor CD200 and CD200R1 and summary CD200/R1 expression by best overall response are shown. **S3A)** Representative images of tumors with no membranous CD200 expression (0), low- (+1), moderate- (+2), and strong- (+3) staining intensity. Overall CD200 expression is calculated as an H-score (histoscore) that is a weighted sum of the staining intensities in the tumor. **S3B)** Representative images of tumors with no- (0%), low- (>1-5%), moderate (6-10%), high (< 11%) CD200R1 positive immune cells. **S3C)** Distribution (median, min, max, Q1, and Q3) of tumor CD200 (H-score) and CD200R1 expression (% of positive immune cells) for participants that received pharmacological doses of 23ME-00610 (> 60 mg) is shown for participants with a best overall response of PR or SD and separately for participants with PD. Mean H-score of 44 (SEM = 21) for CD200 expression was observed in participants that experienced PR/SD (n = 9), compared to a mean H-score of 12 (SEM = 8.7) for participants that progressed on 23ME-00610 treatment. Mean CD200R1 expression in PR/SD participants (n = 8 with evaluable archival tumor tissue) was 4 (SEM = 1.6) vs 4.1 (SEM = 1.1) in participants with PD (n = 10). Solid pink data point represents the pancreatic neuroendocrine patient that achieved a PR with 23ME-00610 treatment. ***Abbreviations***: PR, partial response; SD, stable disease ; PD, progressive disease.

**Supplemental Figure S4. Exploratory Germline Genetic Analysis of European-ancestry First-in-Human Trial Participants Treated with 23ME-00610**

**
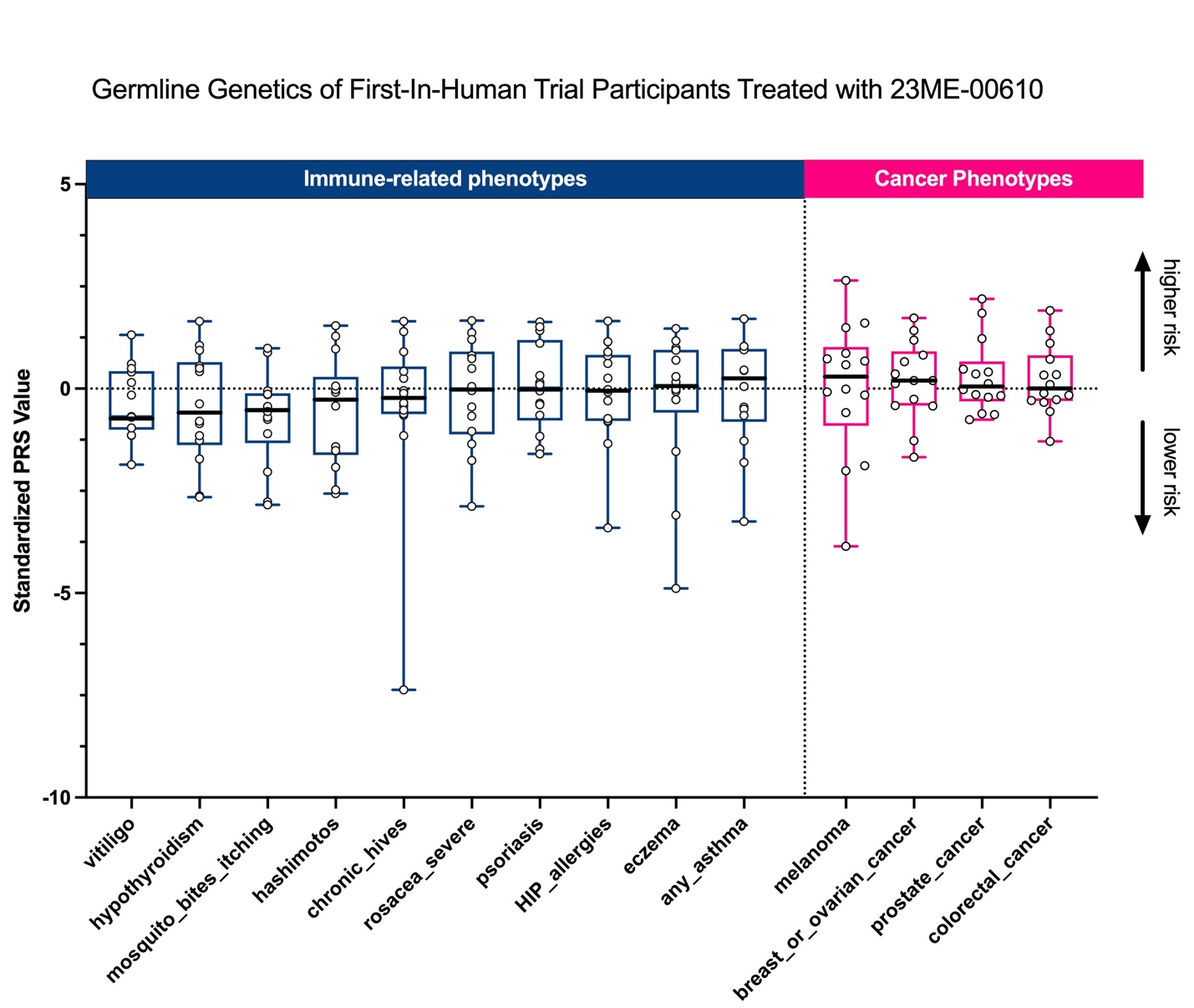
**

**Supplemental Figure 4. Exploratory Germline Genetic Analysis of European-ancestry First-in-Human Trial Participants Treated with 23ME-00610.** Trial participants (n = 14) of self-reported European descent with evaluable genotyping data had PRS values calculated for immune-mediated and cancer phenotypes. The box plot (median, min, max, Q1, and Q3) shows the distribution of the Z-score standardized PRS values of trial participants compared to the mean PRS of the reference European population (dashed line, mean value set at 0).
