# Supplementary material for: First-in-Human Study of 23ME-00610, an Antagonistic Antibody for Genetically Validated CD200R1 Immune Checkpoint, in Participants with Advanced Solid Malignancies
Source: Cancer Res Commun. 2025 Jan 15;5(1):94–105. doi: 10.1158/2767-9764.CRC-24-0568 (PMC11734590; doi:10.1158/2767-9764.CRC-24-0568)
Supplement: Figure S4 — Supplemental Figure S4 [file crc-24-0568_figure_s4_suppsf4.docx]

**Supplemental Figure S4. Exploratory Germline Genetic Analysis of European-ancestry First-in-Human Trial Participants Treated with 23ME-00610**

**
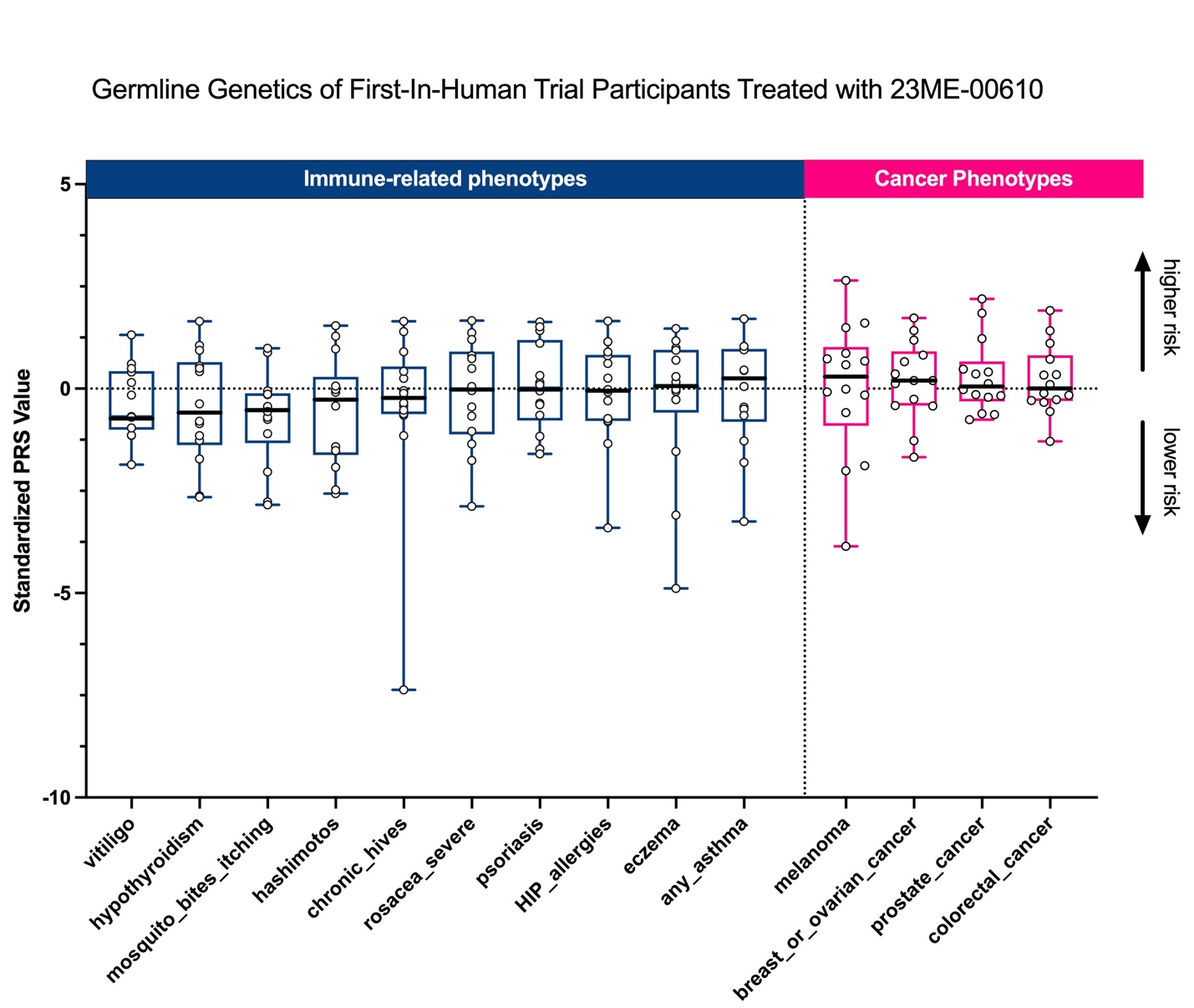
**

**Supplemental Figure 4. Exploratory Germline Genetic Analysis of European-ancestry First-in-Human Trial Participants Treated with 23ME-00610.** Trial participants (n = 14) of self-reported European descent with evaluable genotyping data had PRS values calculated for immune-mediated and cancer phenotypes. The box plot (median, min, max, Q1, and Q3) shows the distribution of the Z-score standardized PRS values of trial participants compared to the mean PRS of the reference European population (dashed line, mean value set at 0).
