# Supplementary material for: First-in-Human Study of 23ME-00610, an Antagonistic Antibody for Genetically Validated CD200R1 Immune Checkpoint, in Participants with Advanced Solid Malignancies
Source: Cancer Res Commun. 2025 Jan 15;5(1):94–105. doi: 10.1158/2767-9764.CRC-24-0568 (PMC11734590; doi:10.1158/2767-9764.CRC-24-0568)
Supplement: Figure S3 — Supplemental Figure S3 [file crc-24-0568_figure_s3_suppsf3.docx]

**Supplemental Figure S3. CD200 and CD200R1 staining intensity and distribution by clinical benefit**

**S3A)
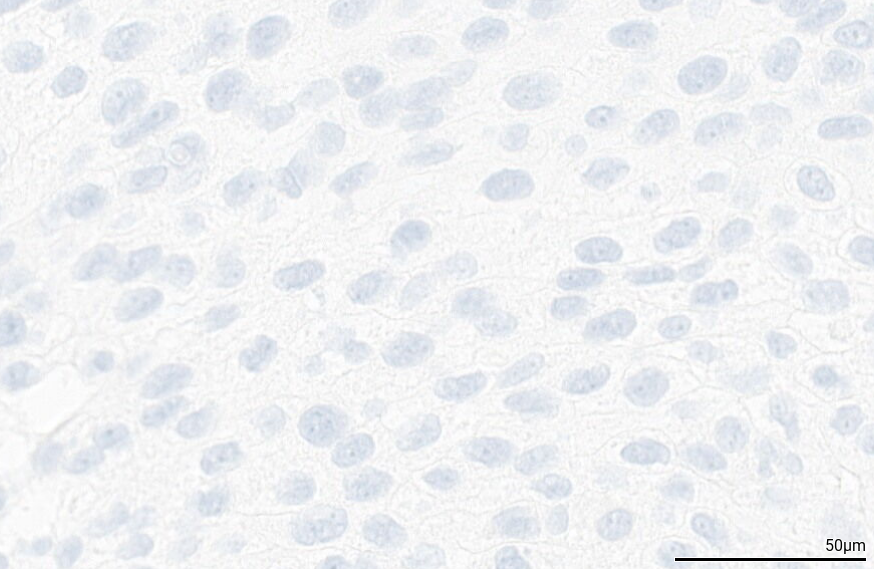
**

**
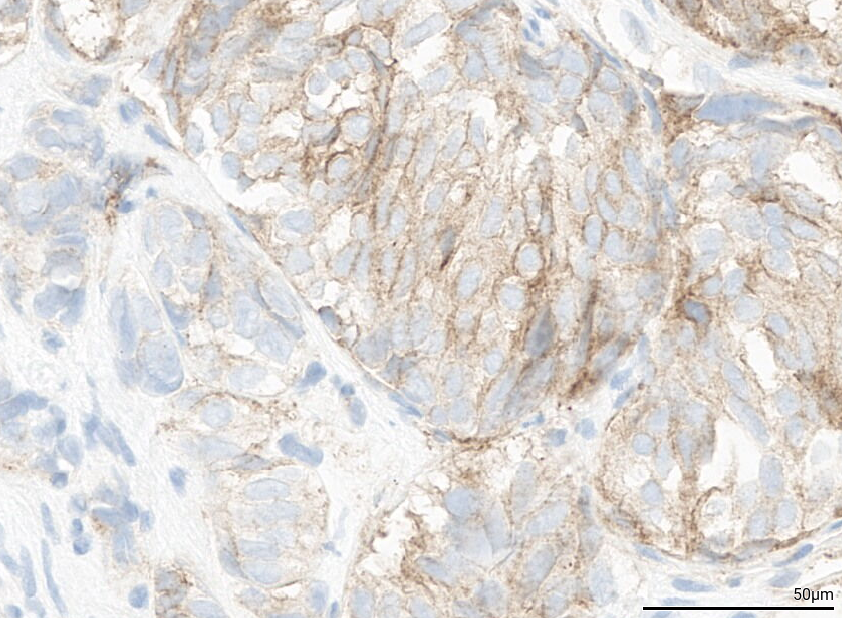
**

**0**

**+1**

**
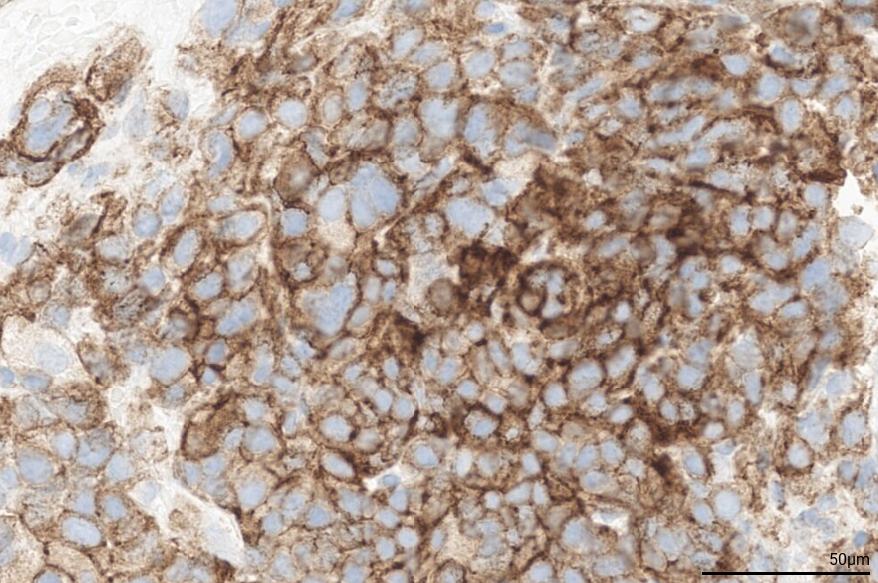

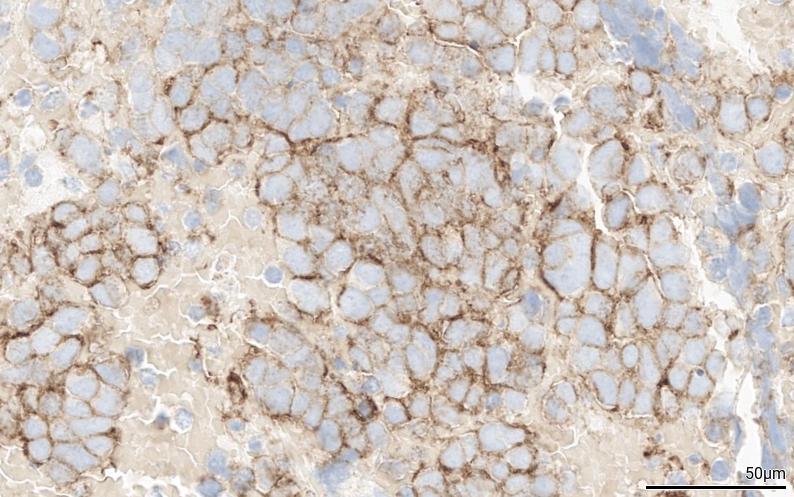
**

**+3**

**+2**

**Supplemental Figure 3A:** Representative images of tumors with no CD200 membranous expression (0), l

**S3B)**

**
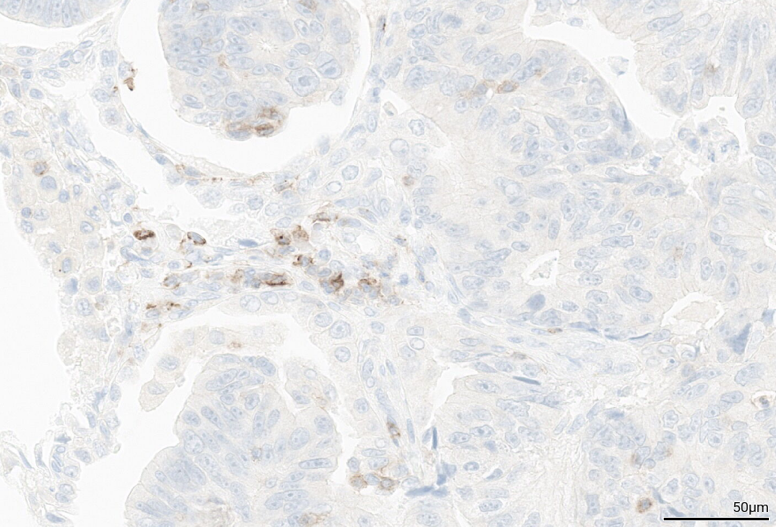

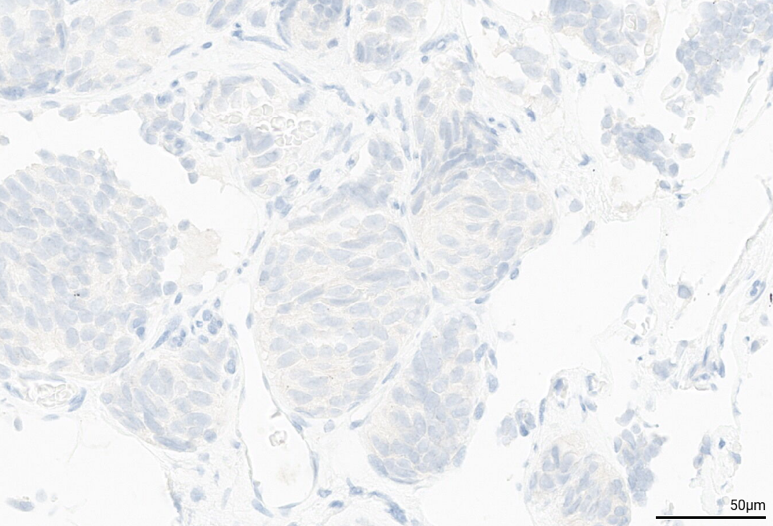
**

**3%**

**0%**

**
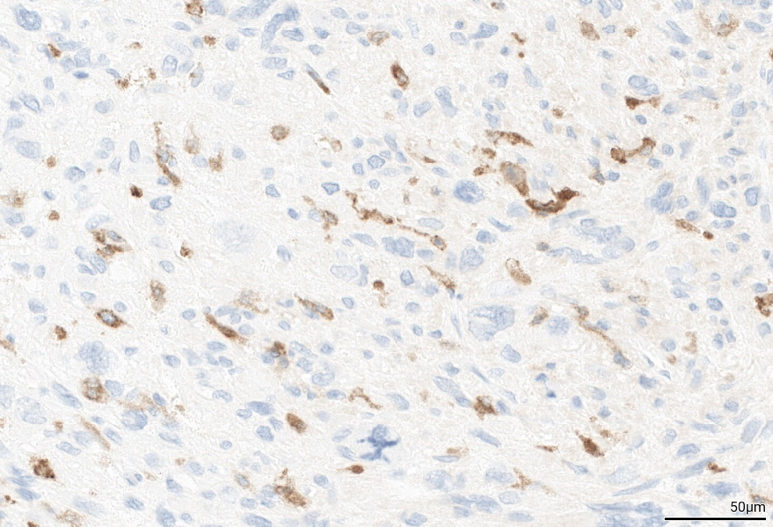

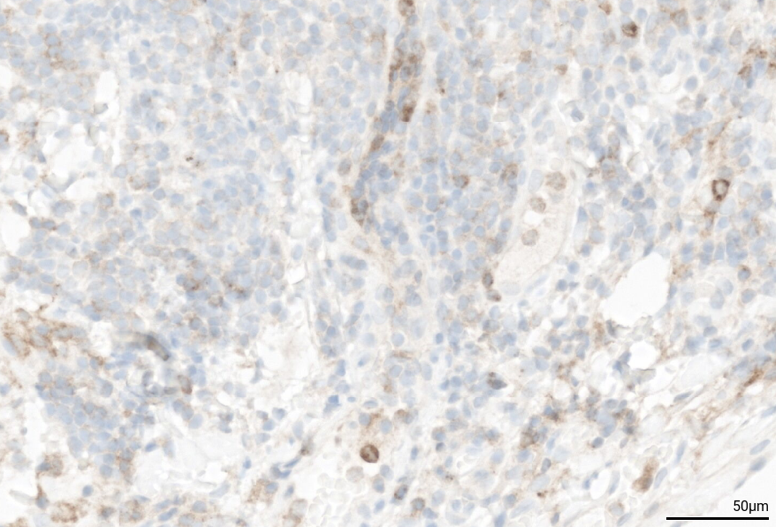
**

**15%**

**8%**

**S3C)**


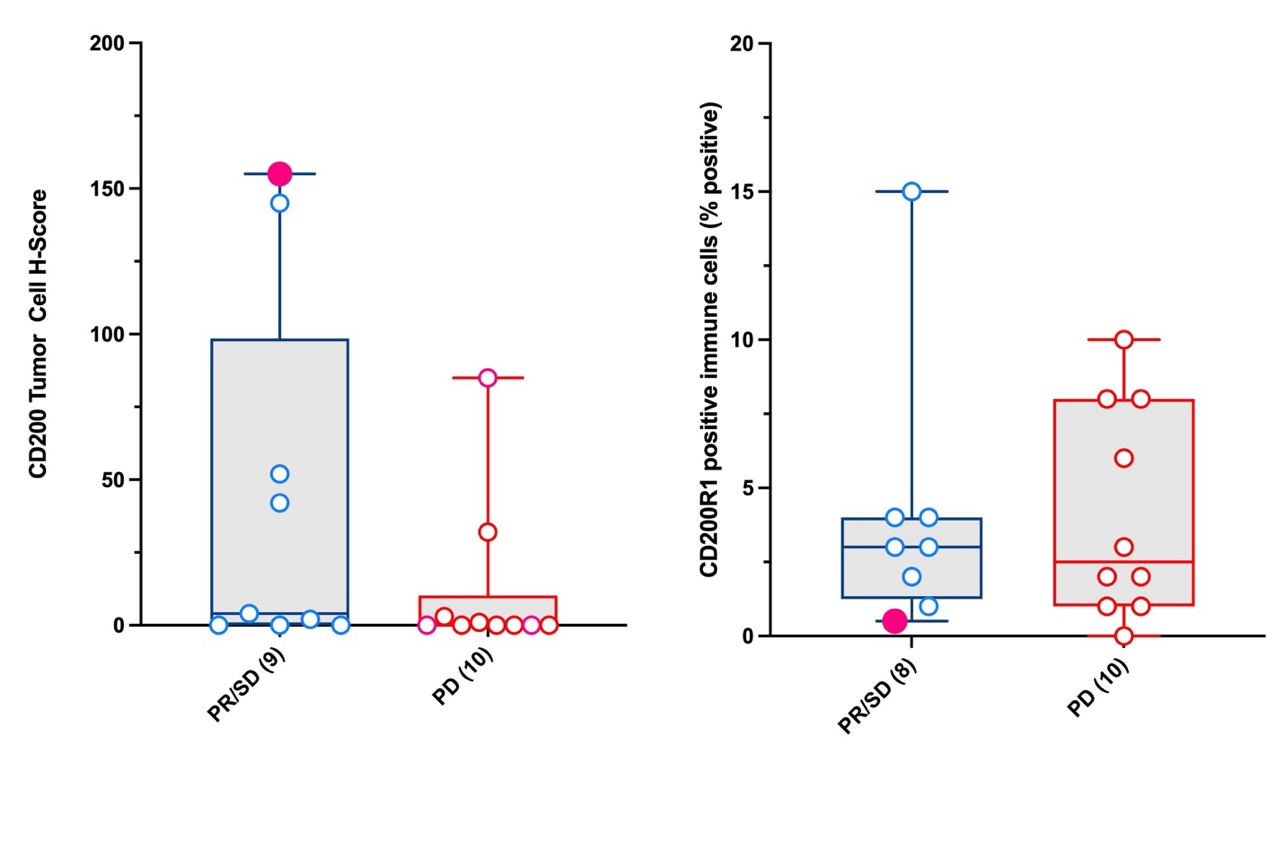


**Supplemental Figure S3.** Representative images of tumor CD200 and CD200R1 and summary CD200/R1 expression by best overall response are shown. **S3A)** Representative images of tumors with no membranous CD200 expression (0), low- (+1), moderate- (+2), and strong- (+3) staining intensity. Overall CD200 expression is calculated as an H-score (histoscore) that is a weighted sum of the staining intensities in the tumor. **S3B)** Representative images of tumors with no- (0%), low- (>1-5%), moderate (6-10%), high (< 11%) CD200R1 positive immune cells. **S3C)** Distribution (median, min, max, Q1, and Q3) of tumor CD200 (H-score) and CD200R1 expression (% of positive immune cells) for participants that received pharmacological doses of 23ME-00610 (> 60 mg) is shown for participants with a best overall response of PR or SD and separately for participants with PD. Mean H-score of 44 (SEM = 21) for CD200 expression was observed in participants that experienced PR/SD (n = 9), compared to a mean H-score of 12 (SEM = 8.7) for participants that progressed on 23ME-00610 treatment. Mean CD200R1 expression in PR/SD participants (n = 8 with evaluable archival tumor tissue) was 4 (SEM = 1.6) vs 4.1 (SEM = 1.1) in participants with PD (n = 10). Solid pink data point represents the pancreatic neuroendocrine patient that achieved a PR with 23ME-00610 treatment. ***Abbreviations***: PR, partial response; SD, stable disease ; PD, progressive disease.
