# Supplementary material for: First-in-Human Study of 23ME-00610, an Antagonistic Antibody for Genetically Validated CD200R1 Immune Checkpoint, in Participants with Advanced Solid Malignancies
Source: Cancer Res Commun. 2025 Jan 15;5(1):94–105. doi: 10.1158/2767-9764.CRC-24-0568 (PMC11734590; doi:10.1158/2767-9764.CRC-24-0568)
Supplement: Figure S2 — Supplemental Figure S2 [file crc-24-0568_figure_s2_suppsf2.docx]

**Supplemental Figure S2. Scans from Screening to Week 24 for Participant with Esophageal Adenocarcinoma**


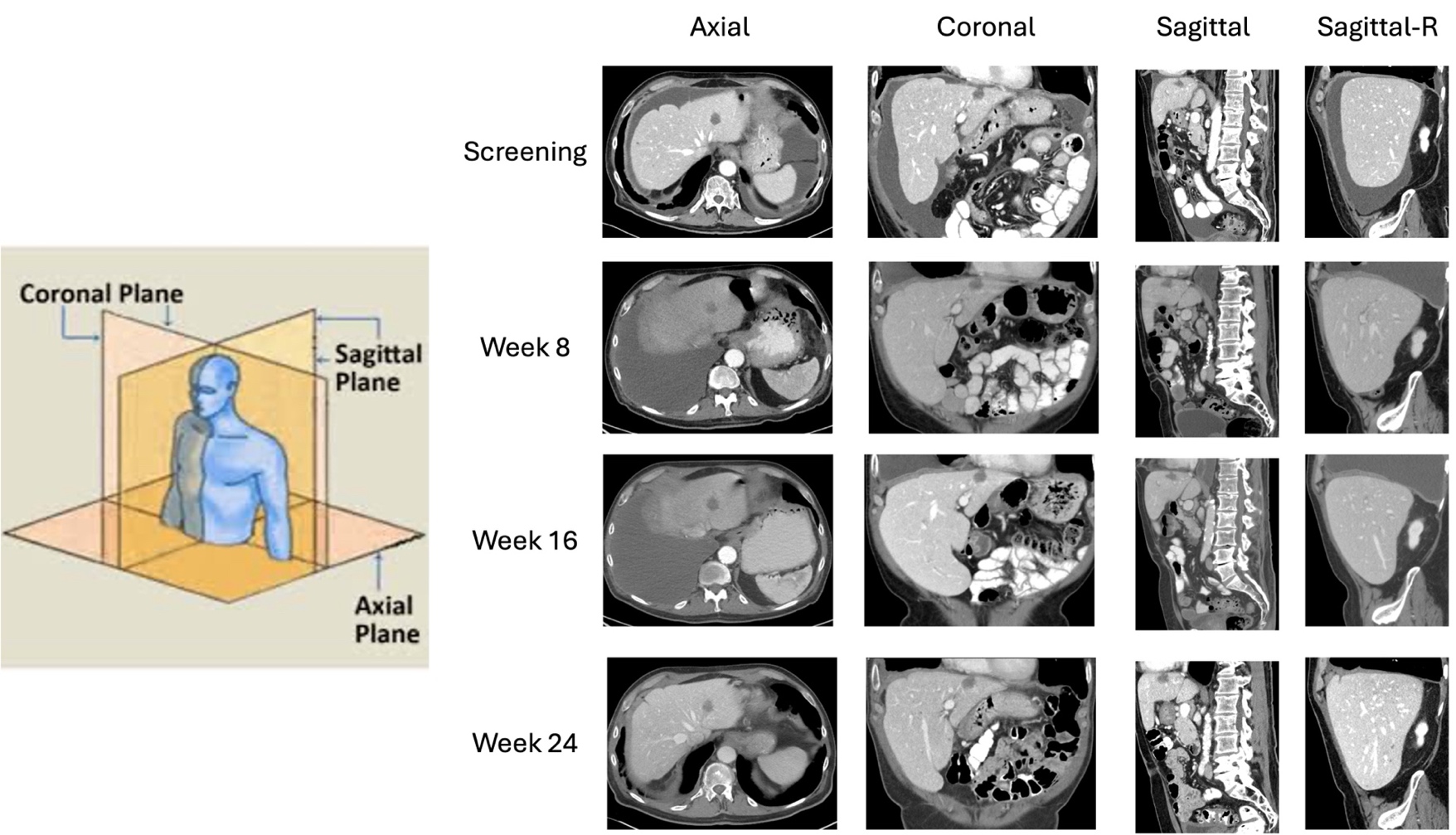


**Supplemental Figure S2.** 66-year old male with esophageal cancer resected in 2019 with recurrent metastatic disease in 2022. Following progression on combination therapy with FOLFOX-nivolumab (FOLFOX = folinic acid, 5-fluorouracil, and oxaliplatin), the patient enrolled on the 23ME-00610 Phase 1 study at the dose escalation dose level of 600 mg administered once every three weeks (Q3W). At baseline (Screening), the patient had liver metastases and ascites. After the first dose, the patient developed an immune related adverse event of rash. Concurrently, the ascites resolved; however, massive right sided pleural effusion developed. With continued 23ME-00610 study treatment, the pleural effusion resolved concurrently with the event of immune related hypothyroidism by Week 24. The patient remains with improved clinical status and without progression of disease after initiating study treatment (> 21 months as of the October 2, 2024 data cutoff). Due to insufficient tumor cell content in the archival tumor sample, tumor CD200 expression was unable to be determined.
