# Supplementary material for: First-in-Human Study of 23ME-00610, an Antagonistic Antibody for Genetically Validated CD200R1 Immune Checkpoint, in Participants with Advanced Solid Malignancies
Source: Cancer Res Commun. 2025 Jan 15;5(1):94–105. doi: 10.1158/2767-9764.CRC-24-0568 (PMC11734590; doi:10.1158/2767-9764.CRC-24-0568)
Supplement: Figure S1 — Supplemental Figure S1 [file crc-24-0568_figure_s1_suppsf1.docx]

**Supplemental Figure S1: Study Design for 23ME-00610 First-in-Human Clinical Trial**

**Supplemental Figure S1.** The Phase 1/2a first-in-human (FIH) multicenter study of 23ME-00610 utilized an accelerated titration followed by "3+3" open-label design. 23ME-00610 was administered by IV infusion every 3 weeks (Q3W) in participants with histologically diagnosed locally advanced (unresectable) or metastatic solid cancer that had progressed after all available standard therapy for the specific tumor type or no further standard therapy existed. The study design included a PK/PD Backfill cohort (N ≤ 12 participants) at levels anticipated to be in the pharmacologically active dose range (ie, ≥ 600 mg).

Abbreviations: IV, intravenously; PK/PD, pharmacokinetics/pharmacodynamics.
